# Contrasting drivers and trends of ocean acidification in the subarctic Atlantic

**DOI:** 10.1038/s41598-021-93324-3

**Published:** 2021-07-07

**Authors:** Fiz F. Pérez, Jon Olafsson, Solveig R. Ólafsdóttir, Marcos Fontela, Taro Takahashi

**Affiliations:** 1 Instituto Investigaciones Marinas (IIM, CSIC), Eduardo Cabello, 6, 36208 Vigo, Spain; 2 Institute of Earth Sciences, University of Iceland, Reykjavik, Iceland; 3 Marine and Freshwater Research Institute, Hafnarfjordur, Iceland; 4 Centre of Marine Sciences (CCMAR), Universidade Do Algarve, 8005-139 Faro, Portugal; 5 Lamont-Doherty Geological Observatory of Columbia University Palisades, Palisades, NY 10964 USA

**Keywords:** Marine chemistry, Climate-change impacts, Biogeochemistry

## Abstract

The processes of warming, anthropogenic CO_2_ (C_anth_) accumulation, decreasing pH_T_ (increasing [H^+^]_T_; concentration in total scale) and calcium carbonate saturation in the subarctic zone of the North Atlantic are unequivocal in the time-series measurements of the Iceland (IS-TS, 1985–2003) and Irminger Sea (IRM-TS, 1983–2013) stations. Both stations show high rates of C_anth_ accumulation with different rates of warming, salinification and stratification linked to regional circulation and dynamics. At the IS-TS, advected and stratified waters of Arctic origin drive a strong increase in [H^+^]_T_, in the surface layer, which is nearly halved in the deep layer (44.7 ± 3.6 and 25.5 ± 1.0 pmol kg^−1^ yr^−1^, respectively). In contrast, the weak stratification at the IRM-TS allows warming, salinification and C_anth_ uptake to reach the deep layer. The acidification trends are even stronger in the deep layer than in the surface layer (44.2 ± 1.0 pmol kg^−1^ yr^−1^ and 32.6 ± 3.4 pmol kg^−1^ yr^−1^ of [H^+^]_T_, respectively). The driver analysis detects that warming contributes up to 50% to the increase in [H^+^]_T_ at the IRM-TS but has a small positive effect on calcium carbonate saturation. The C_anth_ increase is the main driver of the observed acidification, but it is partially dampened by the northward advection of water with a relatively low natural CO_2_ content.

## Introduction

The oceanic absorption of anthropogenic CO_2_ (C_anth_) is causing major changes in marine carbonate chemistry^1^. The ocean generally remains slightly basic; however, C_anth_ uptake increases the concentration of total hydrogen ions ([H^+^]_T_), decreasing the pH_T_ and the concentration of carbonate ions [CO_3_^2−^]. This is collectively referred to as ocean acidification (OA^2,3^), and it affects calcifying marine organisms^4,5^. The seasonality and decadal trends of OA in surface waters across heterogeneous oceanic regions have been summarized on the basis of a handful of consolidated fixed stations^6^. The future impact of OA will depend on variations in the long-term mean and on the short-term temporal variability of carbonate chemistry^7,8^. Few observational studies address OA in the deep ocean in subarctic regions^9–15^, where OA is predicted to influence ecosystems early^4,16,17^.

The Atlantic Meridional Overturning Circulation (AMOC) plays a major role in climate change attenuation, making the North Atlantic (NA) one of the strongest ocean sinks for heat, natural carbon^18^ and C_anth_^19–22^. The NA accumulates more than 30% of the heat absorbed^23^ and over 25% of the C_anth_ accumulated in the ocean. At the millennial scale, this circulation also favours the spreading of deep-sea cold-water corals in the Atlantic, but their near future is threatened by the deep transport of acidified seawater^17^. Deep convection in subarctic regions plays a key role in understanding AMOC variability^24^.

The extreme weather conditions of subarctic regions make it difficult to achieve long time series with enough frequency to characterize the seasonal cycle and, consequently, to determine the long-term trends and interannual variability. Several studies have observed divergent trends with the expected increase in CO_2_ in the atmosphere^25–27^ depending on the time frame of the observations. In the subpolar NA, McKinley et al*.*^28^ find that the long-term trends in surface pCO_2_ take 25 years to emerge from changes due to variability on a decadal scale and converge with the atmospheric trend.

**Figure 1:**
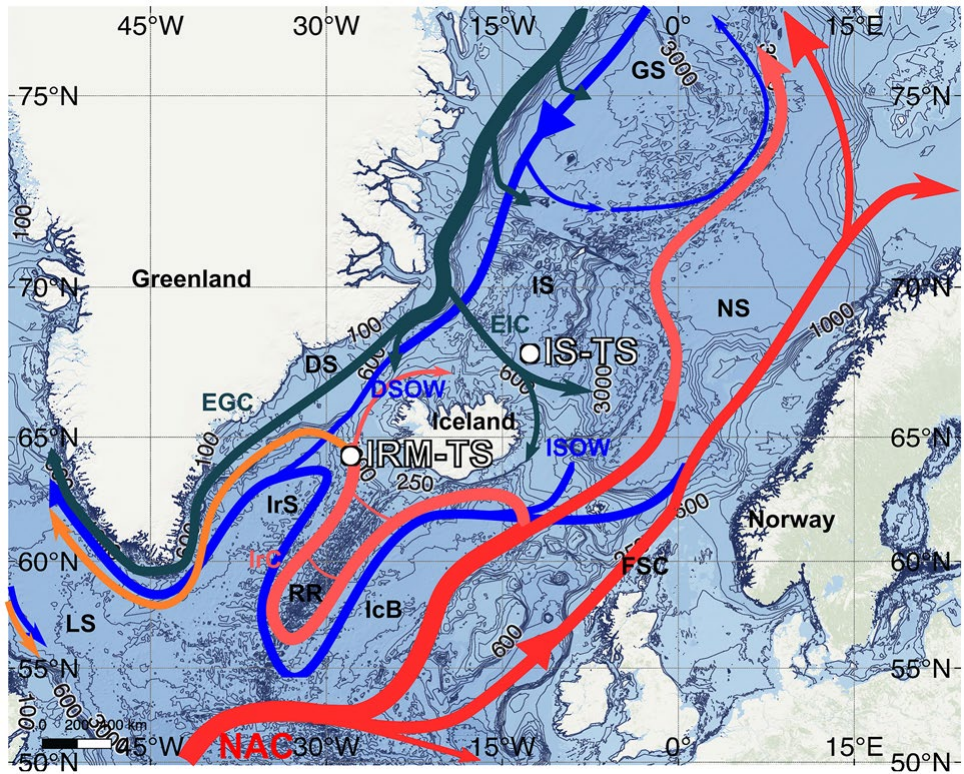
Map of the subarctic Atlantic showing the main surface and deep currents. The locations of the Iceland fixed station (IS-TS) and Irminger fixed station (IRM-TS) described in this study are indicated by white dots. A schematic diagram of the large-scale circulation of the main water masses is shown: North Atlantic Current (NAC), Irminger Current (IrC), East-Icelandic Current (EIC), East Greenland Current (EGC) Denmark Strait Overflow (DSOW) and Iceland Scotland Overflow Water (ISOW). Some geographical features are also shown: Labrador Sea (LS), Irminger Sea (IrS), Iceland Basin (IcB), Greenland Sea (GS), Iceland Sea (IS), Norwegian Sea (NS), Reykjanes Ridge (RR), Denmark Strait (DS) and Faroe-Scotland Channel (FSC). Schematic diagram of the large-scale circulation is adapted from Våge et al.^34^ and Lherminier et al.^36^.

From 1983 to present, the Icelandic Marine Research Institute (MRI) in cooperation with Lamont-Doherty Earth Observatory (LDEO) monitored hydrographic conditions quarterly at two stations located in the Iceland Sea (IS-TS; 68.0°N, 12.67°W) and in the Irminger Sea (IRM-TS; 64.33°N, 28.0°W). This large, high-quality database included in GLODAPv2^29^ has allowed the study of the variability in the marine carbonate system and the assessment of OA trends^10,30,31^. The surface waters at IS-TS are under the influence of the low-salinity East-Icelandic Current flowing to the southeast along with some contribution from the modified waters of the NA flowing northwards (NAC, Fig. 1). In intermediate layers, the thermohaline properties at IS-TS are essentially Arctic Intermediate Waters (AIW) located above the maximum temperature (0.8 °C) of the deep waters of the Arctic^32–34^. The IRM-TS is located within the Irminger Current, which is the eastern branch of the warm and saline North Atlantic Current, where the mixed layer depth (MLD) reaches a thickness of more than 300 m in winter and is dominated by subpolar mode waters^23,31,35–37^ and by Labrador Sea water (LSW) coming from the SW region of the Irminger Sea^36,38^. The stations present clear hydrographic contrasts, which are reflected in the thermohaline characteristics of the water masses (see Supplementary Fig. S1). Despite this, both stations are relatively well ventilated, showing similarly low values of oxygen undersaturation (low apparent oxygen utilization (AOU), see Supplementary Fig. S2). This is a common feature in the NA, where deep layer ventilation is generally high. Both the oceanographic characteristics of the two stations and the high quality of the dataset allow us to evaluate the long-term evolution of OA and to analyse the different drivers of [H^+^]_T_ and [CO_3_^2−^] from the surface mixed layer to the deep layers. This study shows that there are contrasting drivers acting at both sites by the decadal variability of oceanographic conditions and the CO_2_ increase.

## Results

### Mean hydrographic vertical variability

**Figure 2:**
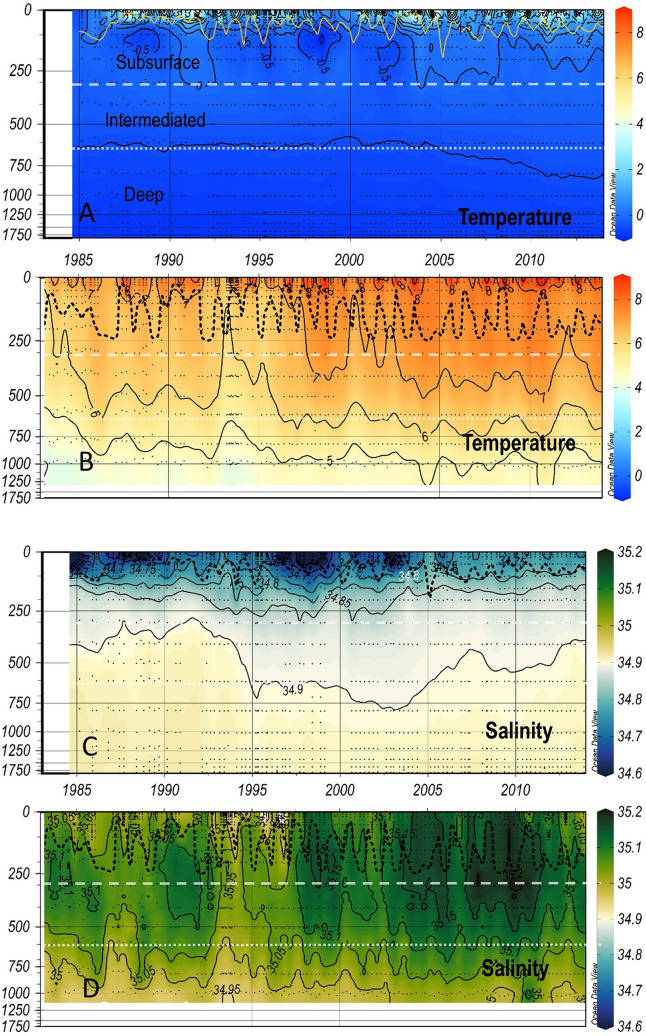
Vertical and time variation from 1983 (1985 for the IS-TS) to 2013 of temperature and salinity (black isolines and colour scale) at the IS-TS (**A**,**C**) and at the IRM-TS (**B**,**D**). The dashed black line shows the mixed layer depth that delimits the surface from the subsurface layers. The white dashed line separates the subsurface and intermediate layers, and the white dotted lines separate the intermediate and deep layers (labelled in **A**). Figure prepared using Ocean Data View/DIVA^69^ 5.4.0 http://odv.awi.de.

**Figure 3:**
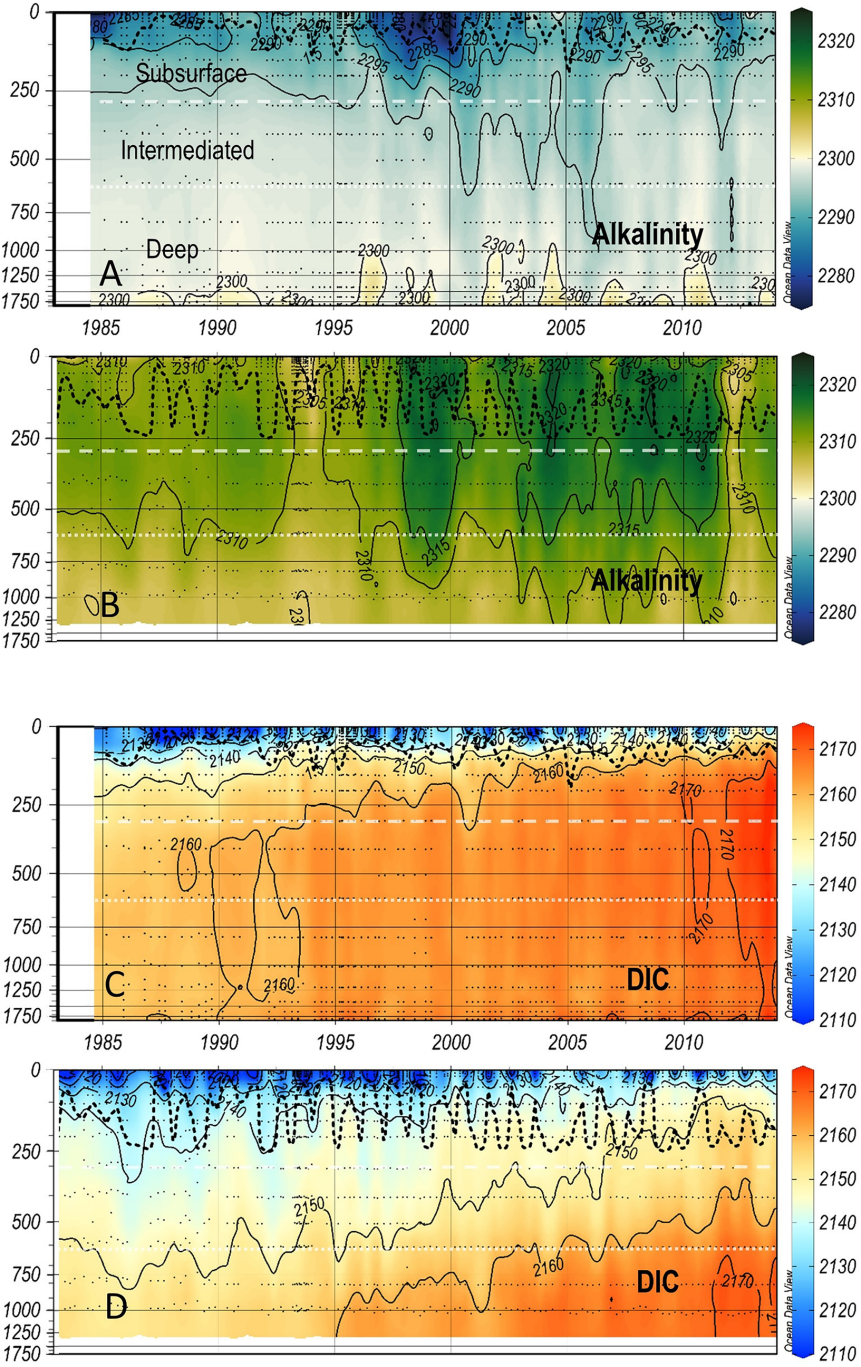
As Fig. 2 for alkalinity and DIC at IS-TS (**A**,**C**) and at IRM-TS (**B**,**D**).

**Figure 4:**
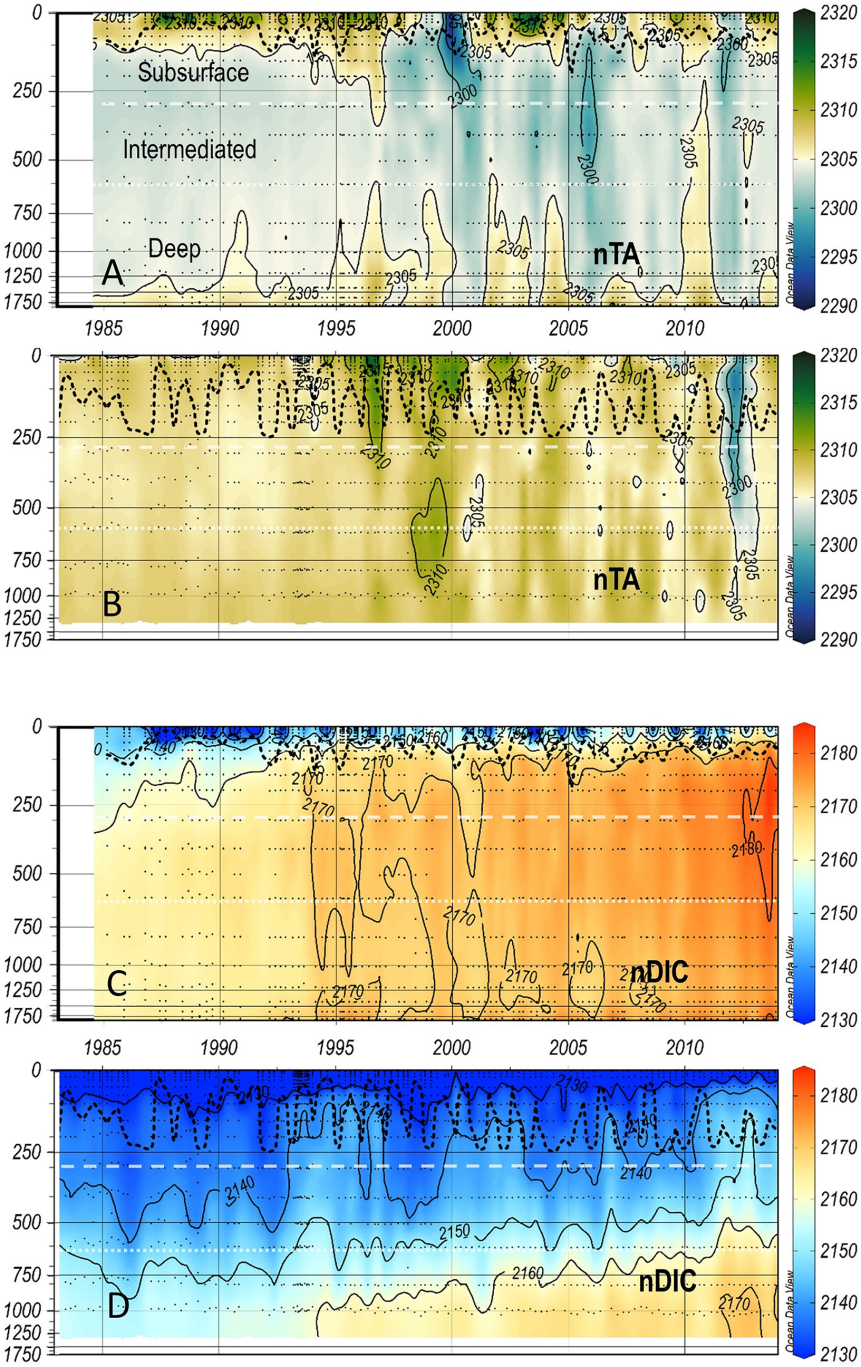
As Fig. 2 for salinity-normalised total alkalinity (nTA) and DIC (nDIC) at IS-TS (**A**,**C**) and at IRM-TS (**B**,**D**).

**Figure 5:**
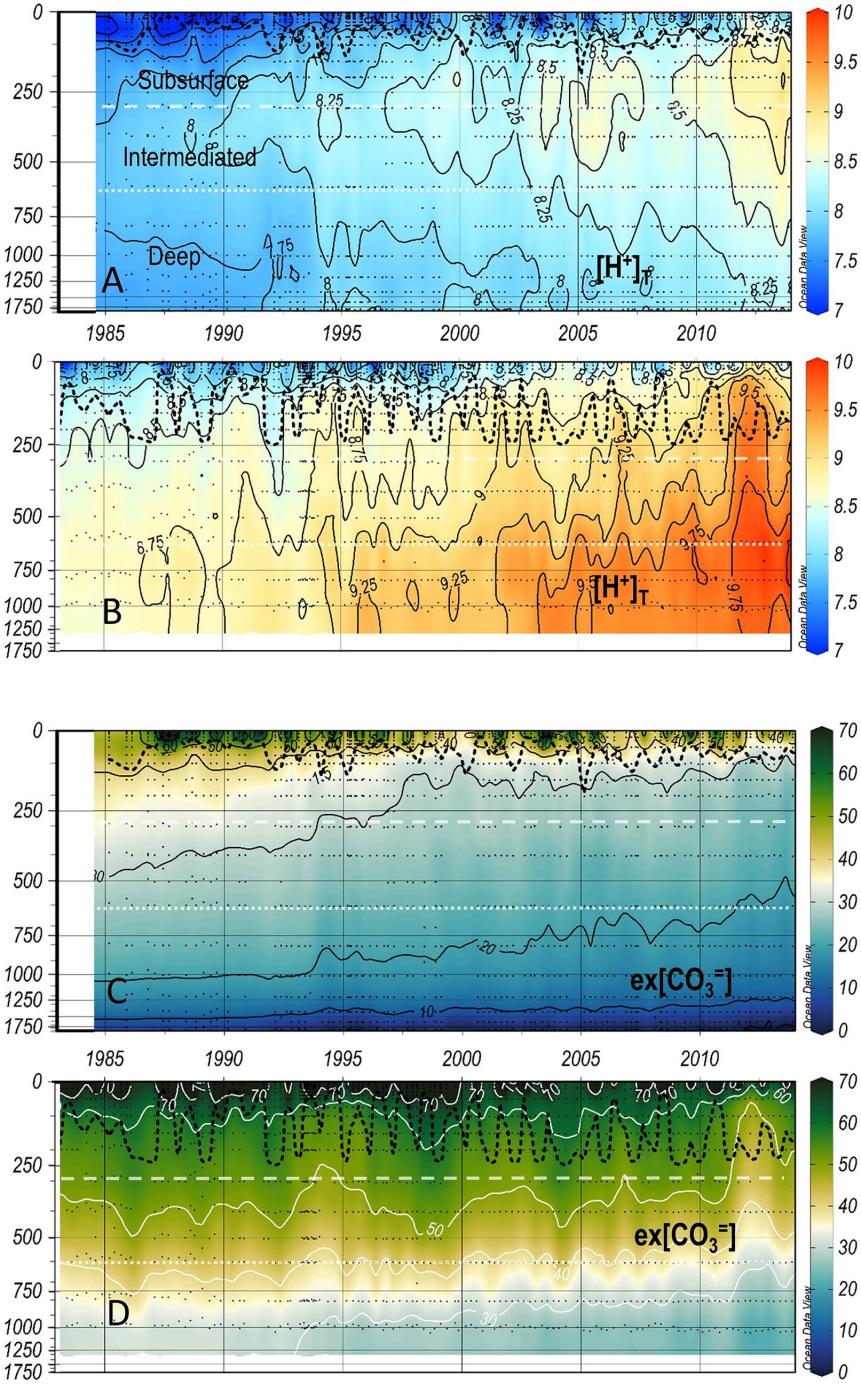
As Fig. 2 for total hydrogen ions concentration in nmol/kg (**[**H^+^]_T_) and the excess of [CO_3_^=^ ] over the [CO_3_^=^ ] at aragonite saturation in µmol/kg (ex[CO_3_^=^]) at IS-TS (**A**,**C**) and at IRM-TS (**B**,**D**).

Based on the watermass structure, four layers have been defined that summarize the vertical variability at the two stations (see methods). The surface layer ranges from the surface to the depth of the MLD following the criterion adopted by Jeansson et al*.*^39^ for the IS-TS, which shows strong seasonal variability (black dashed line in Figs. 2, 3, 4 and 5). In winter, the in situ temperature (T) and salinity (S) profiles show a mixed surface layer that is typically 200 m thick (Fig. 2a) with smooth variations with depth and smooth transitions from intermediate to deep waters. At the IRM-TS, the winter MLD can reach a thickness of 300 m, driving a strong reduction in the thickness of the subsurface layer (from the MLD to 300 m). The intermediate layer, between 300 and 600 m, is characterized by a rather smooth vertical salinity gradient dominated by different varieties of subpolar mode water (SPMW). Below 600 m, the deep layer is characterized by the thermohaline gradient generated by mixing with LSW (see Supplementary Fig. S1).

Three decades of temperatures clearly show differences at both stations (Fig. 2). The average T in the four layers are distinctly different (Table 1). This same contrast is also shown in the salinity values^34,38^. Both T and S observed at the IS-TS are lower than those recorded at the IRM-TS. At the IRM-TS, a frequent S maximum is observed in the subsurface layer associated with SPMW. With similar thermal gradients, the vertical saline gradient observed at the IRM-TS station is weaker than that observed at the IS-TS station, which favours winter mixing and deepening of the MLD. Analogous to S, the total alkalinity (TA) shows low values at the IS-TS, showing a relatively weak vertical gradient with low variability at both stations (Fig. 3, Table 1). The normalization of alkalinity to salinity 35, nTA (= TA*S/35)^27^, homogenizes both the differences between the stations and their vertical gradients, especially at the IRM-TS. This indicates that TA variability is practically determined by S. The variability in dissolved inorganic carbon (DIC) is very similar at both stations. In the deep layers, their mean values are indistinguishable with a very low standard deviation. In the upper layers, DIC is slightly higher at the IS-TS, although both stations present high variability. With the normalization to a salinity of 35, nDIC (nDIC = DIC*S/35)^27^ shows that water masses at the IS-TS contain a higher nDIC, possibly due to a higher solubility of CO_2_ at low temperatures, which is partially compensated at the IRM-TS by its higher salinity (Fig. 4). The pH_T_ values (at in situ temperature and pressure) are consistently higher throughout the water column at the IS-TS, with the highest values in the surface layer but with a lower vertical gradient than at the IRM-TS station, which has the lowest values in the deep layer (Fig. S2). Since both TA and DIC are very similar at both stations, the lower temperature at the IS-TS determines their higher pH_T_ values. Consequently, this is inversely transferred to [H^+^]_T_ (Fig. 5, see Methods). When pH_T_ refers to the same T of 4 °C (Supplementary Fig. S3), the pH_T_ (4 °C) values are lower at the IS-TS than at the IRM-TS. At the same T, a decrease in pH_T_ implies a thermodynamic decrease in [CO_3_^=^] and a consequent decrease in the excess [CO_3_^=^] (ex[CO_3_^=^]) over [CO_3_^=^ ] at aragonite saturation. The decrease in pH_T_(4 °C) is consistent with the lower values in ex[CO_3_^=^] in the surface layer at the IS-TS with respect to the IRM-TS and explains 80% of the observed difference. The lower T at the IS-TS also leads to a lower ex[CO_3_^=^] of approximately 15%. This difference is maintained in the rest of the profile, with a parallel decrease in both, motivated in part by the T and pH_T_(4 °C) decrease, and by the pressure increase that generates an increase in aragonite solubility, thereby decreasing the difference between [CO_3_^=^] and aragonite saturation, ex[CO_3_^=^ ] (Fig. 5). At the IS-TS, the average values of C_anth_ show a clear vertical gradient, with high values in the surface layer gradually decreasing to approximately one-third of those values in the deep layer, where the highest AOU indicates less ventilation^20,40,41^. In contrast, at the IRM-TS, the values are high and very homogeneous in all layers (Supplementary Fig. S4).

**Table 1:** Average and standard deviations of the in situ temperature (T in °C), salinity (S), Total Alkalinity (TA in µmol kg^−1^), salinity-normalized alkalinity (nTA in µmol kg^−1^), total dissolved inorganic carbon (DIC in µmol kg^−1^), salinity-normalized dissolved inorganic carbon (nDIC in µmol kg^−1^), in situ pH in total scale (pH_T_), total hydrogen ion concentrations ([H^+^]_T_ in nanomol kg^−1^), ion carbonate concentration excess over aragonite saturation (exCO_3_^=^ in µmol kg^−1^), and anthropogenic CO_2_ (C_anth_ in µmol kg^−1^).

Layer	Stn	T	S	TA	nTA	DIC	nDIC	pH	[H^+^]_T_	exCO_3_	C_anth_
Surface	IS	2.4 ± 3.0	34.69 ± 0.10	2289 ± 7	2309 ± 7	2125 ± 28	2144 ± 26	8.124 ± 0.036	7.57 ± 0.62	52.4 ± 19.5	46 ± 11
0 to MLD	IRM	7.89 ± 1.68	35.06 ± 0.08	2312 ± 6	2308 ± 5	2119 ± 29	2115 ± 27	8.096 ± 0.042	8.10 ± 0.79	71.8 ± 18.6	48 ± 9
Subsurface	IS	−0.02 ± 0.31	34.84 ± 0.03	2293 ± 3	2304 ± 3	2158 ± 8	2168 ± 7	8.087 ± 0.020	8.33 ± 0.39	32.0 ± 4.2	37 ± 9
MLD to 300 m	IRM	6.91 ± 0.68	35.10 ± 0.06	2314 ± 4	2307 ± 3	2145 ± 6	2138 ± 5	8.057 ± 0.017	8.94 ± 0.37	54.4 ± 4.0	49 ± 9
Intermediate	IS	−0.24 ± 0.08	34.89 ± 0.01	2296 ± 2	2303 ± 2	2165 ± 5	2171 ± 5	8.081 ± 0.013	8.62 ± 0.27	26.5 ± 2.7	30 ± 7
300–600 m	IRM	6.91 ± 0.68	35.10 ± 0.06	2314 ± 4	2307 ± 3	2145 ± 6	2138 ± 5	8.057 ± 0.017	8.94 ± 0.37	54.4 ± 4.0	47 ± 9
Deep	IS	−0.77 ± 0.05	34.91 ± 0.00	2299 ± 2	2305 ± 2	2165 ± 4	2170 ± 4	8.096 ± 0.011	8.99 ± 0.23	12.8 ± 2.1	19 ± 4
600-bottom	IRM	5.15 ± 0.45	35.02 ± 0.03	2309 ± 3	2307 ± 2	2159 ± 7	2158 ± 6	8.038 ± 0.019	9.83 ± 0.43	33.1 ± 3.5	39 ± 8

### Contrasting acidification trends

Table 2 shows the average trends obtained with the seasonally detrended data (see Methods) of pH_T_, [H^+^]_T_ and ex[CO_3_^=^], as well as additional parameters which drive acidification trends for the complete periods of each of the two stations. The p-values and r^2^ are also included. For comparative purposes, the same trends were also obtained using only the original data observed in the winter months (Supplementary Table S1). The trends are not significantly different, and in general, a better accuracy in the estimations is obtained using the full set of seasonally detrended data (see Methods).

**Table 2:** Average trends obtained with the seasonally detrended data the in situ temperature (T in °C yr^−1^), salinity (S in yr^−1^), Total Alkalinity (TA in µmol kg^−1^ yr^−1^), salinity-normalized alkalinity (nTA in µmol kg^−1^ yr^−1^), total dissolved inorganic carbon (DIC in µmol kg^−1^ yr^−1^), salinity-normalized dissolved inorganic carbon (nDIC in µmol kg^−1^ yr^−1^), in situ pH in total scale (pH_T_ yr^−1^), total hydrogen ion concentrations ([H^+^]_T_ in picomol kg^−1^ yr^−1^), ion carbonate concentration excess over aragonite saturation (exCO_3_^=^ in µmol kg^−1^ yr^−1^), and anthropogenic CO_2_. The errors of the trends are included. Intercalated rows show p-level and R^2^ in parenthesis.

Layer	St	T	S	TA	nTA	DIC	nDIC	pH_T_	[H^+^]_T_	exCO_3_^=^	C_anth_
Surface	IS	0.034 ± 0.009	0.004 ± 0.001	0.15 ± 0.07	−0.14 ± 0.05	0.94 ± 0.13	0.68 ± 0.11	−2.58 ± 0.22	44.7 ± 3.6	−0.443 ± 0.080	1.15 ± 0.06
		0.06 (0.12)	0.03 (0.21)	0.17 (0.04)	0.13 (0.06)	0.02 (0.31)	0.03 (0.24)	0.0070 (0.56)	0.0064 (0.58)	0.03 (0.21)	0.0029 (0.75)
	IRM	0.063 ± 0.005	0.004 ± 0.001	0.22 ± 0.04	−0.04 ± 0.04	0.49 ± 0.10	0.24 ± 0.10	−1.70 ± 0.19	32.6 ± 3.4	−0.125 ± 0.070	0.93 ± 0.03
		0.006 (0.58)	0.02 (0.30)	0.03 (0.19)	0.54 (0.01)	0.04 (0.16)	0.16 (0.05)	0.0124 (0.40)	0.0110 (0.43)	0.27 (0.03)	0.0009 (0.90)
Subsurface	IS	0.014 ± 0.002	**0.001 ± 0.000**	0.03 ± 0.03	−0.03 ± 0.02	0.78 ± 0.03	0.73 ± 0.03	−2.26 ± 0.08	43.2 ± 1.6	−0.417 ± 0.022	1.00 ± 0.03
		0.03 (0.22)	0.09 (0.09)	0.67 (0.01)	0.41 (0.02)	0.0019 (0.82)	0.0021 (0.80)	0.0013 (0.87)	0.0014 (0.87)	0.0027 (0.77)	0.0010 (0.90)
	IRM	0.050 ± 0.003	0.004 ± 0.000	0.22 ± 0.03	−0.02 ± 0.02	0.49 ± 0.03	0.27 ± 0.04	−1.50 ± 0.08	31.6 ± 1.7	−0.134 ± 0.030	1.00 ± 0.03
		0.0044 (0.65)	0.012 (0.40)	0.0207 (0.28)	0.70 (0.01)	0.0037 (0.69)	0.02 (0.27)	0.0026 (0.76)	0.0028 (0.75)	0.05 (0.14)	0.0008 (0.91)
Intermediate	IS	0.002 ± 0.001	0.000 ± 0.000	−0.04 ± 0.02	−0.01 ± 0.02	0.52 ± 0.02	0.54 ± 0.02	−1.52 ± 0.06	30.2 ± 1.2	−0.303 ± 0.013	0.70 ± 0.04
		0.12 (0.06)	0.08 (0.10)	0.30 (0.02)	0.90 (0.00)	0.0019 (0.82)	0.0017 (0.84)	0.0016 (0.84)	0.0017 (0.84)	0.0018 (0.83)	0.0033 (0.73)
	IRM	0.043 ± 0.003	0.003 ± 0.000	0.18 ± 0.03	−0.01 ± 0.02	0.51 ± 0.03	0.33 ± 0.04	−1.54 ± 0.07	32.9 ± 1.6	−0.162 ± 0.028	0.90 ± 0.03
		0.0058 (0.59)	0.011 (0.42)	0.03 (0.24)	0.89 (0.00)	0.0034 (0.71)	0.0128 (0.39)	0.0022 (0.79)	0.0024 (0.78)	0.03 (0.22)	0.0012 (0.88)
Deep	IS	0.005 ± 0.000	0.000 ± 0.000	−0.04 ± 0.02	−0.02 ± 0.02	0.39 ± 0.02	0.41 ± 0.02	−1.23 ± 0.05	25.5 ± 1.0	−0.231 ± 0.010	0.38 ± 0.03
		0.0015 (0.86)	0.01 (0.41)	0.23 (0.03)	0.75 (0.01)	0.0029 (0.75)	0.0027 (0.76)	0.0016 (0.85)	0.0016 (0.84)	0.0019 (0.82)	0.0050 (0.64)
	IRM	0.028 ± 0.003	0.002 ± 0.000	0.13 ± 0.02	0.00 ± 0.02	0.71 ± 0.02	0.58 ± 0.03	−1.95 ± 0.04	44.2 ± 1.0	−0.306 ± 0.018	0.73 ± 0.04
		0.012 (0.42)	0.012 (0.41)	0.03 (0.23)	1.00 (0.00)	0.0009 (0.90)	0.0020 (0.81)	0.0005 (0.94)	0.0005 (0.94)	0.0033 (0.71)	0.0029 (0.74)

Both stations show significant and widespread warming. At the IS-TS, the warming is highest in the surface layer and decreases rapidly in the intermediate layer. However, weak warming in the deep layer is clearly visible by the downward displacement of the isotherm of − 0.5 °C (Fig. 2a) after 2004. Warming in all layers at the IRM-TS is significantly higher, almost doubling the trends observed at the IS-TS. S also shows increasing, significant and generalized trends at the IRM-TS, while at the IS-TS, only two shallower layers show positive trends. In the deep layers at the IS-TS, the 34.9 isohaline showed a decadal oscillation with high values between 1993 and 2007.

At the IS-TS, the TA trends are not significant in any of the layers. The same is true for nTA. In contrast, significant positive DIC trends occur at both stations and at all levels (Table 2). The TA at the IRM-TS shows significant positive trends in all layers, consistent with the observed salinification trends (Table 2, Fig. 3). These trends are very similar in the entire water column. Once normalized, the nTA (Fig. 3) trend practically disappears, indicating that the balance of freshwater inputs and/or the advection of more saline waters can explain the observed changes in TA^27^. At the IS-TS, the maximum DIC trends are found in the surface layer (Fig. 3), decreasing towards the bottom following the expected penetration of C_anth_ (Supplementary Fig. S4) from the sea surface. In fact, their trends follow the same pattern of C_anth_ trends but are somewhat lower. In contrast, the trends in the surface and subsurface layers at the IRM-TS are significantly lower than those observed at the IS-TS and lower than the trends of the C_anth_ increase. Interestingly, the deeper layer at the IRM-TS shows the highest increasing DIC trend that corresponds to the expected value of the C_anth_ increase. Normalization of DIC suggests that the salinity effect on DIC trends at the IS-TS is not significant, except for the surface layer, where a lower trend in nDIC is found, suggesting that 30% of the change in DIC is due to salinity. Considering the mean coarse ratio DIC:S = 2150:35, a salinity trend of 0.004 yr^−1^ would suppose a change in DIC of 0.24 µmol kg^−1^ yr^−1^, which is consistent with the differences between DIC and nDIC trends. At the IRM-TS, the saline effect in the DIC trend is much more marked, with significantly lower trends in nDIC than in DIC. This shows that very important changes in DIC are due to salinity changes due to the advection of warm, saline waters with relatively lower values of DIC^26,42,43^ associated with the strengthening of the North Atlantic Current^36,43,44^. Although the trend of nDIC in the surface layer at the IRM-TS is low and not significant, the values obtained with winter data are significant at 90% (p-level = 0.08; Table S1).

Both stations show significant acidification trends in terms of pH_T_, [H^+^]_T_ and ex[CO_3_^=^. The maximum absolute trends are observed in the surface layer at the IS-TS in all three variables. The subsurface layer at the IS-TS shows a slightly smaller decreasing trend in pH_T_ but is indistinguishable in [H^+^]_T_ and ex[CO_3_^=^] from the surface trends. In the intermediate and deep layers, the trends are clearly lower and are the lowest of both stations. The vertical gradient, therefore, has decreasing absolute values and is higher than that observed at the IRM-TS with a lower vertical gradient. Here, contrary to what is expected, the absolute values of the acidification trends are maximum in the deep layer. The absolute trends are lower but very similar in the subsurface and intermediate layers.

### Acidification drivers

**Figure 6:**
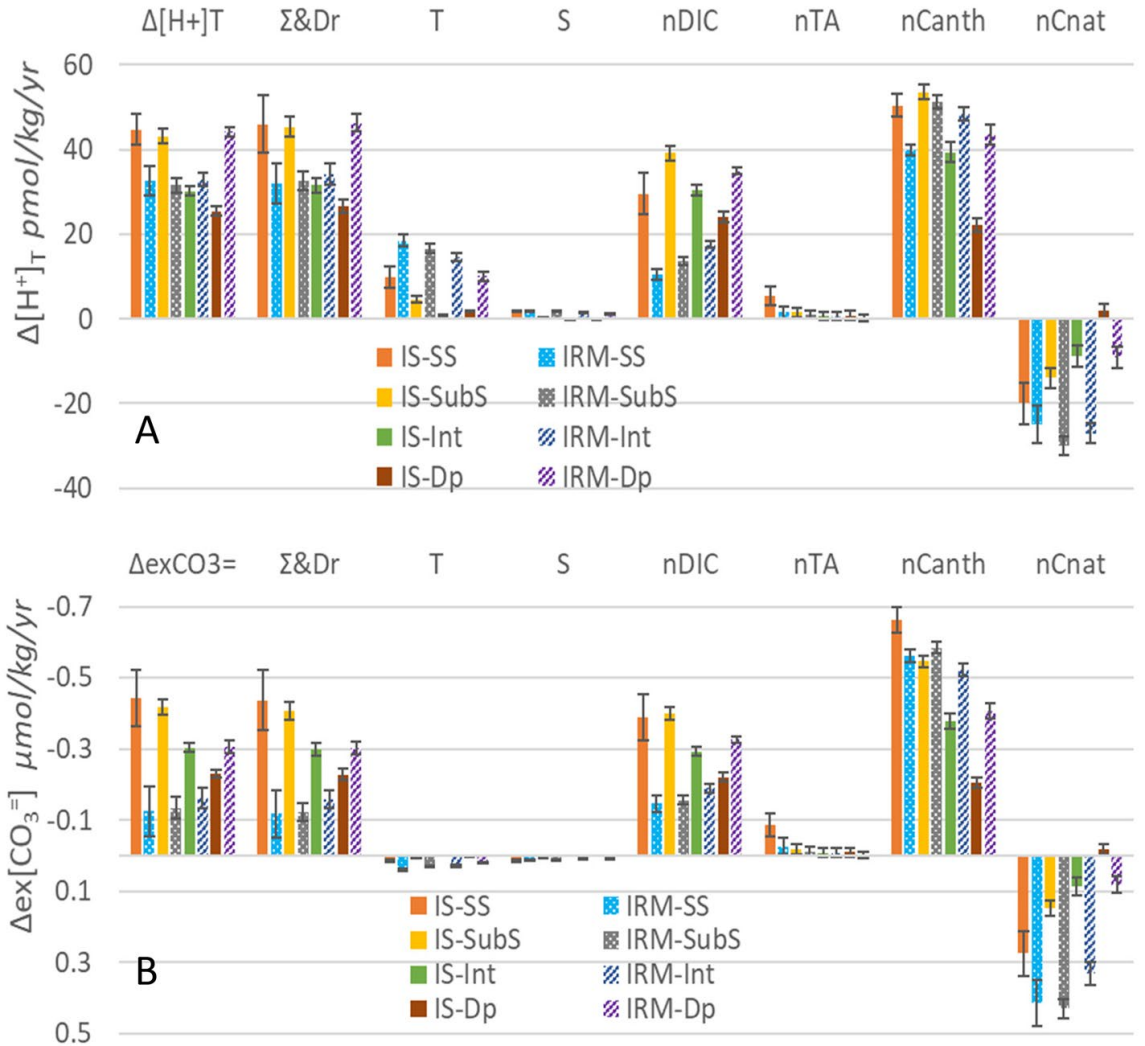
Acidification trends and drivers decomposition (T,S, nDIC and nTA) for the seasonally detrended average time series of total hydrogen ions concentration in pmol/kg/yr (Δ**[H**^**+**^**]**_**T**_, **A**) and for excess of [CO_3_^=^ ] over the [CO_3_^=^ ] at aragonite saturation in µmol/kg/yr (Δ**ex[CO**_**3**_^**=**^**]**, **B**). The nDIC driver trends is split in natural (nCnat) and anthropogenic components (nCanth). The colour code is shown on both panels.

The regional OA trends ([H^+^]_T_ and ex[CO_3_^=^]) at both fixed stations are presented in Fig. 6 for seasonally detrended time series and in Supplementary Table S1 and Fig. S5 for original data. Considering that the surface layer of both stations was kept in equilibrium with the atmosphere and taking the air CO_2_ concentrations registered in the island station located in Iceland (Storhofdi, Vestmannaeyjar, 63.40°N, 20.29°W)^45^, the annual trends of DIC, pH_T_, [H^+^]_T_ and ex[CO_3_^=^] can be estimated for reference. At the IS-TS (IRM-TS), the expected annual trends of ocean acidification would be 1.93 (1.98)·10^−3^ of pH_T_ per year, 39 (38) pmol/kg/yr of [H^+^]_T_ and 0.44 (0.52) µmol/kg/yr of ex[CO_3_^=^], assuming that T, S, TA remain constant at the mean values given in Table 1 for the surface layer. These trends would be accompanied by an increase in DIC or nDIC of 0.78 (0.86) µmol/kg/yr at the IS-TS (IRM-TS). The estimated trends, although similar, are different between the two stations (Table 2). At the IS-TS, the trends are somewhat higher, except for an undiscernible trend in ex[CO_3_^=^]. In contrast, at the IRM-TS, the absolute trends are significantly lower. Deviations from the atmospheric reference indicate that other factors or drivers are involved^26,44^.

The impact of small linear changes in S and nTA on the increase in [H^+^]_T_ is positive, and some of them are significant. Both drivers together reach a maximum value of 16% of the change in [H^+^]_T_ in the surface layer at the IS-TS and decrease rapidly towards the deep layer. At the IRM-TS, these drivers sum from 7% on the surface to 2% in the bottom layers. In relative terms, T is the second driver favouring the increase in [H^+^]_T_. At the IS-TS, its incidence is relatively high in the surface layer (22%), decreasing in the rest of the water column. Much more relevant is its impact at the IRM-TS. In the surface and subsurface layers, it exceeds the impact of the increase in nDIC with an incidence of more than 50%. In the two deeper layers, its impact is reduced to 23% in the deep layer. Therefore, the high trends of warming have a significant impact on the increase in [H^+^]_T_.

The increase in nDIC is the dominant driver at the IS-TS, with a relative impact of 66% on the surface layer and greater than 85% in the rest of the water column. In contrast, at the IRM-TS, the increase in nDIC explains only 32 and 42% of the increase in [H^+^]_T_ in the shallower layers, while its impact is clearly predominant in the deep layer (75%). The separation of DIC changes between natural and anthropogenic components allows us to discern their impact on [H^+^]_T_ trends. In general, the C_anth_ component drives high trends of acidification, even higher than those expected from an increase in equilibrium with the atmosphere (approximately 40 µmol/kg)^15^, except for the deep layer at the IS-TS, where it contributes 83% of the increase in [H^+^]_T_. At the IRM-TS, the influence of C_anth_ is higher than 100%, compensating for the negative effect of the natural component (nC_nat_) and weakening the increase in [H^+^]_T_ between 20 and 95% in the subsurface layer. The sum of all the drivers (T, S, nDIC and nTA) reproduces the observed trends with an error of less than 5% with somewhat greater uncertainty than the trend obtained directly from [H^+^]_T_.

Similarly, the drivers of the ex[CO_3_^=^] were evaluated (Fig. 6b). As happens for [H^+^]_T_, the effects of T and S are very reduced at the IS-TS, although with a tendency to favour the increase in ex[CO_3_^=^] with maximum values of 7% together in the surface layer, which opposes the TA (− 19%) with an large uncertainty. In IRM-TS, as in the case of [H^+^]_T_, the impact of T and S is stronger and in combination they favour the increase of ex[CO_3_^=^] with a combined value of 32% of the final value, being reduced only to a 10% in the deep layer. Although the trends in nTA are very low, it has a minor impact on the decrease of ex[CO_3_^=^] (only if the surface layer at the IS-TS reaches 20%). The dominant driver that promotes the decrease in ex[CO_3_^=^] is the increase in nDIC in all layers and at both stations, corresponding practically to the observed trends of ex[CO_3_^=^]. The percentage at the IRM-TS ranges from 107 to 118% of the resulting trend and overcompensates for the joint favourable effects of T and S. At the IS-TS, the impact ranges from 88% in the surface layer to 98% in the rest of the water column, while the small contributions of T, S and nTA cancel each other out. The partition between natural and anthropogenic nDIC is consequently parallel to that described for [H^+^]_T_.

## Discussion

Located outside the main ocean currents of the NA, both stations similarly undergo the warming and long-term salinification observed during the last decades^23,43,46,47^. The IRM-TS shows a long-term surface warming of 0.063 ± 0.005 (Table 2), which is consistent with those shown recently by Leseurre et al*.*^25^ (0.05 ± 0.01 C yr^−1^ between 1993 and 2017) in the northern Irminger Sea. The lower warming trend shown by these authors is due to the recent cooling period between 2008 and 2017 (− 0.05 ± 0.01 per year), which was also observed by Frob et al*.*^27^. In the same region and time period, a similar long-term trend of salinification (0.005 ± 0.001 yr^−1^) was also observed by Leseurre et al*.*^25^, which is consistent with interannual freshening episodes (Fig. 2). The observed maximum salinity at 300 m in 2010 suggests the advection of thick saline seawater from the south^26,42,43^. In contrast, the low S period 1993–1997 is related to an eastward extension of the subpolar gyre^48^. The Nordic Seas, mainly due to the advection of warmer and saltier surface waters of the NA through the Faroes-Shetland Islands Channel, showed warming and salinification in the upper 2000 m to 2016^43,46^. Between 1985 and 2017, similar trends of surface and subsurface annual warming and salinification were shown by Lauvset et al*.*^43^ (0.025 ± 0.005 C yr^−1^ and 0.002 ± 0.0003 yr^−1^ in the upper 500 m layer of the Greenland Sea; see their Fig. 2). However, the presence of low-salinity waters on the western margin of the Nordic Seas with the presence of AIW seems to prevent warming and salinification in the deep layers at the IS-TS. Only a sharp positive shift in temperature and salinity between 1998 and 2000 has been related to circulation changes in the Icelandic region^49^. In contrast, the weak stratification in the Irminger Sea allows warming and salinification to occur at high rates in the deep layer, with values similar to those observed for Labrador Sea Water of 0.025 ± 0.002 °C yr^−1^ and 0.00334 ± 0.00024 yr^−1^ by Garcia-Ibañez et al*.*^14^. In summary, the physical drivers, namely, warming and salinification, show a consistent pattern in both basins and with some similarities, despite the strong thermohaline differences, with the main contrast being the strong impact of the warming on the deep layers at the IRM-TS.

The acidification trends found here in the surface layer at the IS-TS and IRM-TS are within the values observed in other time series collected by Bates and others^6^. The trend of pH_T_ decrease at the IRM-TS (1.70 ± 0.19 10^−3^ yr^−1^) is very close to that determined by Leseurre et al*.*^25^ in the northern Irminger Sea (1.6 ± 0.1 10^−3^ yr^−1^ using winter data) and to that determined by Garcia-Ibañez et al*.*^14^ in the same basin from hydrographic sections of the A-25 GOSHIP line from 1991 to 2015 (1.8 ± 0.1 10^−3^ yr^−1^), while Vázquez-Rodríguez et al*.*^11^ obtained the same trend between 1981 and 2008 for the same section. Lauvset et al*.*^50^ found a similar trend of 2.0 ± 0.4 10^−3^ yr^−1^ for the North Atlantic subpolar seasonally stratified (NA-SPSS) biome from 1991 to 2011. All these trends contrast with the high acidification trend of 2.37 ± 0.49 10^−3^ yr^−1^ reported by Bates et al*.*^6^ using the same database and associated with an increase in DIC of 1.62 ± 0.35. The acidification trend estimated here for the IS-TS is among the highest trends globally, 2.58 ± 0.22 10^−3^ yr^−1^, and is consistent with that determined by Olafsson et al*.*^9^ of 2.4 ± 0.2 10^−3^ yr^−1^ between 1985 and 2008 using only winter data associated with an increase in surface DIC of 1.44 ± 0.23 µmol kg^−1^ yr^−1^. These trends are significantly larger than those reported for the same period by Bates et al*.*^6^ (1.4 ± 0.5 10^−3^ yr^−1^) associated with an increase in DIC of 1.22 ± 0.27 µmol kg^−1^ yr^−1^. Apparently, an exchange of those Iceland and Irminger trends reported by Bates et al*.*^6^ in their Table 2 would be much more comparable with the trends described here, in addition to those obtained by Olafsson et al*.*^9^, Vázquez-Rodríguez et al*.*^11^, Lauvset et al*.*^50^, Garcia-Ibañez et al*.*^14^ and Leseurre et al*.*^25^. In the subarctic western North Pacific, the winter mixing layer (K2 and KNOT time series stations) showed significantly lower acidification trends (0.80 ± 0.4 10^−3^ yr^−1^) for the 1997–2011^12^ period, which was attributed to the damping generated by a strong increasing trend in TA. This has led to a much slower long-term decline in pH_T_ in this region than that observed at any other time series stations in the open ocean^6^ and in NA subpolar gyre for 1993–2017 (Table 2).

Some aspects related to the non-linearity of the pH_T_ scale should be highlighted. The surface layer at the IS-TS and the deep layer at the IRM-TS present the same trend of acidification in terms of [H^+^]_T_; however, the absolute trend in pH_T_ units is 25% higher at the IS-TS, where the mean pH_T_ observed is almost 0.1 units higher than in the deep layer at the IRM-TS (Table 1). Deep layers, usually with a lower pH_T_, show higher acidification changes if expressed in [H^+^]_T_ than on the logarithmic scale^7,8^. Proportionally to the trends in the surface layer, the trends in the deep layer of [H^+^]_T_ are higher than those for pH_T_. In fact, the trend of reduction of pH_T_ in the deep layer is 45% with respect to the surface, while in terms of [H^+^]_T_, it is only reduced to 55%. Therefore, it is better to express the acidification trends in [H^+^]_T_, avoiding the non-linearity of the logarithmic scale^7,51^ and because seawater pCO_2_ has a much more linear (99.5%) relationship to [H^+^]_T_ than to pH_T_^8^. The effects of pressure and temperature on CaCO_3_ solubility induce a reduction in the vertical correlation between the ex[CO_3_^=^] and [H^+^]_T_ (or of pH_T_) trends.

No trends of ex[CO_3_^=^] reduction have been reported, and very few have been reported in terms of reduction of aragonite saturation (Ω_arag_). The Ω_arag_ trends can be converted to an ex[CO_3_^=^] trend multiplied by the saturation concentration (approx. 66 µmol kg^−1^). Consequently, the decreasing trend of ex[CO_3_^=^] at the IS-TS is very similar to that reported by Olafsson et al*.*^9^ (0.46 ± 0.05 µmol kg^−1^ yr^−1^) at the same station for the period of 1985–2008 and that assigned to the IRM-TS of 0.53 ± 0.26 µmol kg^−1^ yr^−1^ in Bates et al.^6^. These trends contrast with the low trends obtained here for the IRM-TS, except in its deepest layer. The surface trend at the IS-TS is lower than that at the other time-series stations in the subtropical region of the Atlantic Ocean (0.76 ± 0.15 µmol kg^−1^ yr^−1^, ESTOC station; 0.63 ± 0.05 µmol kg^−1^ yr^−1^, BATS station) or even the equatorial region of the Pacific (0.56 ± 0.07 µmol kg^−1^ yr^−1^, HOT station). From climatology carbonic system data, Jiang et al*.*^52^ determined average profiles of ex[CO_3_^=^] reduction in the North Atlantic for 1989–2010 from 0.16 µmol kg^−1^ yr^−1^ at the surface to 0.33 µmol kg^−1^ yr^−1^ to 500 m, with a reduction between 700 and 1000 m to 0.07–0.12 µmol kg^−1^ yr^−1^. The profiles observed at the IRM-TS show some similarity with the average profile described by Jiang et al*.*^52^, while at the IS-TS, the ex[CO_3_^=^] reduction trends are somewhat higher. Olafsson et al*.*^9^ reported a decrease of − 0.080 ± 0.002 µmol kg^−1^ yr^−1^ for waters deeper than 1,500 m at the IS-TS during 1985–2008, which is consistent with our results. The decrease in Ω_arag_ in the LSW layers corresponds to a shoaling of the aragonite saturation horizon (ASH) at an average trend of 10.0 ± 0.4 m yr^−1^, which is in agreement with the 10–15 m yr^−1^ estimated at the centre of the Irminger Sea during 1991–2016^17^. All these values are clearly lower than the high decreasing trend of ex[CO_3_^=^] given by Murata et al*.*^53^ in the first 400 dbar of the subtropical South Pacific during 1994–2009 (1.65 µmol kg^−1^ yr^−1^).

The trends observed in C_anth_ show high surface values, even with higher than expected values assuming equilibrium with the atmosphere^15^. This high trend observed in the surface layer at the IS-TS is consistent with the decrease in pCO_2_ disequilibrium observed by Fransner et al*.*^15^. Notably, the high trends observed in deep layers at the IRM-TS are consistent with other observations^20–22^ in the Irminger Sea or in the North Atlantic subpolar gyre. At the IS-TS, the deep layer has much lower trends that are consistent with less ventilation^20,40,41^. The temporal evolution of C_anth_ at both stations shows quite different patterns. At the IS-TS, the continuous increase in C_anth_ in the surface that is attenuated at depth suggests an invasion from the surface layer. In contrast, penetration at the IRM-TS is much more intense, showing a gradual increase in thick layers of more than 500 m. This suggests that winter convection and lateral advection of SPMW that recirculate cyclonically from the east are the mechanisms promoting the high accumulation of C_anth_^19,54^_._

Except for the surface layer at the IS-TS, where a small negative trend in nTA is detected that promotes negative trends in exCO_3_, the variability of TA is almost entirely determined by S, suggesting that changes in TA due to biogeochemical effects of the CaCO_3_ pump are not appreciable.

Lauvset et al*.*^50^ performed a decomposition of the drivers of the global surface pH_T_ trends in different biomes. The IRM-TS and IS-TS are included in the NA-SPSS biome defined by Lauvset et al*.*^50^. They found that the long-term increase in DIC in this biome is by far the dominant driver of long-term changes in pH_T_, with no temperature effect and a small attenuating contribution to the pH_T_ decrease due to the increase in TA and S. These results are similar to those obtained by Garcia-Ibañez et al*.*^14^ following the same methodology from observations made in the southern Irminger Basin. All of this is consistent with what is observed here at the IS-TS in the increase of [H^+^]_T_; however, for the IRM-TS in this study, we find that T has a very relevant impact. Additionally, Garcia-Ibañez et al*.*^14^ evaluated the drivers in the whole water column by watermass layers. The dominant driver was the increase in DIC, which was mainly due to the anthropogenic component. However, in the LSW layer, the temperature-driven pH_T_ change contributes 25% of the pH_T_ change, close to what is found here (23% in the deep layer of IRM-TS). In addition, Garcia-Ibañez et al*.*^14^ showed that the natural component of DIC had an important contribution to the decrease in pH_T_ in LSW, while in SPMW, the change is attenuated by a decrease in C_nat_. This is in agreement with the findings here, where natural DIC contributes to attenuating both the increase in [H^+^]_T_ and the decrease in ex[CO_3_^=^]. This is consistent with the increased advection of saline waters from the south with lower nDIC^23,43,46,47^. The contrasts between the IS-TS and IRM-TS also refer to the negative C_nat_ changes that attenuate OA. While at the IS-TS, decreases in AOU (increased ventilation) lead to a decrease in C_nat_, at the IRM-TS, it is the advection of warmer, saltier waters with low DIC:Alk ratios that support the decrease in C_nat_. The drivers representing warming, long-term freshwater divergence, or an increase in atmospheric CO_2_ have been defined, and their effects on each of the fixed stations are modulated by their own dynamic characteristics. Contrasting drivers of ocean acidification at these subarctic stations are not only due to differences in the thickness of the winter mixed layer but also dynamic causes. The advection of saline waters within the upper limb of the AMOC brings waters with a low C_nat_ component that are saturated in C_anth_. Even the strengthening of the circulation in the upper limb of the AMOC^26,43,46^ introduces saline waters with low nDIC in the NA and partly buffers OA associated with CO_2_ uptake. On the other hand, on the north side, increased ventilation decreases C_nat_, attenuating OA associated with CO_2_ uptake. In fact, a large part of the natural DIC (C_nat_) trends showed some correlation with the T trends, which suggests several combined effects of warming in addition to thermodynamic effects^26^.

Detailed analysis of the drivers, including the changes in DIC and TA due to S increase, as well as the warming and salinification drivers, improves the analysis because it separates the impact due to physical processes that change the salt balance from the biogeochemical processes that affect nDIC and nTA changes^27^. The dynamics associated with increased advection of saline and low nDIC waters seem to attenuate the pH_T_ declines due to increased C_anth_. Therefore, warming appears to have an intensifying effect on the increase in [H^+^]_T_ at both stations, especially at the IRM-TS, where it becomes preponderant in the surface layer. In contrast, the effect of temperature on aragonite saturation has a small attenuating effect on the impact of the DIC increase^52^.

## Conclusions

The existing certainties of ocean acidification are supported by a handful of fixed stations located mainly in warm waters with high buffering capacities. In contrast, polar and sub-polar waters with lower buffering capacities and lower natural pH values would reach aragonite undersaturation before the end of the century, which affects calcareous organisms. The two stations studied here, which are located in the subarctic region of the NA, showed contrasting acidification trends. In the Icelandic Sea, there are very high trends in the surface layer (44.7 ± 3.6 pmol kg^−1^ yr^−1^ of [H^+^]_T_), which decrease rapidly at depth. In the Irminger Sea, the acidification trend was maximal in deep waters with similar trends (44.2 ± 1.0 pmol kg^−1^ yr^−1^ of [H^+^]_T_).

The impact of the decrease in aragonite saturation levels (or ion carbonate excess) was less than that expected from the increase in [H^+^]_T_ because warming slightly compensated for the negative effects of the increase in DIC while reinforcing the increase in [H^+^]_T_. This is evident at the surface of the Irminger station, where the warming observed in the study period (1983–2013) was very high (0.063 ± 0.005 °C yr^−1^), which contributed 50% to the increase in [H^+^]_T_.

The driver analysis suggests that changes associated with the transport of key climate change properties such as temperature, salinity and DIC by the upper AMOC have contrasting effects on OA in the subarctic Atlantic. Warming has a very marked effect on the increase in [H^+^]_T_ in the surface layers of the Iceland and Irminger Seas and very small positive effects on carbonate saturation. The gradual increase in anthropogenic DIC transport^15^ in the surface layer of the Iceland Sea and in deep and thick layers of the Irminger Sea promotes both an increase in [H^+^]_T_ and a decrease in carbonate saturation. However, changes in saline water transport with low DIC/alkalinity ratios reduce the impact of the increased anthropogenic component. The presence of Arctic origin waters in the deep Iceland Sea dampens the changes observed in the surface layer.

## A tribute to TaroTakahashi

The two time-series of observational data discussed here would not have been produced without Taro Takahashi. The beginning was a cooperative programme between the LDEO and the MRI in Reykjavik. It was intended to be a seasonal investigation, and first observations were in March 1983. Two seasonal cycles revealed large ΔpCO_2_ variability which was in contrast with the general view of the region being an intense CO_2_ sink throughout the year^55^. Therefore, another year of observations was added and further years followed which soon gave the data series value, describing distinct characteristics of the North Atlantic regions^30^. The samples collected were initially shipped to LDEO for analysis. Milestones were reached in 1991 and 1993 when instruments from Lamont for TCO_2_ and pCO_2_ determinations came to MRI with support from Taro and Icelandic research funds. This increased the capacity from surface layer to whole water column observations.

The materialistic outline above does not explain cooperation which lasted for decades. The key element there was Taro´s modest wisdom and deep knowledge which he shared in an atmosphere of equality. His responses to notes on data and interpretations were always detailed and constructive. This spirit of cooperation was likewise felt in exchanges with Taro´s able LDEO technical personnel.

## Methods (1500)

### Data sets

The Icelandic MRI carried out continuous hydrographic sampling consisting of quarterly cruises (February, May, August and November) from 1983 to 2013. The two stations are located at 64.33°N, 28.0°W (IRM-TS) with a depth of 1000 m, and at 68.0°N, 12.67°W (IS-TS) with a depth of 1850 m. The database, methodologies and quality control are well described and detailed by Olafsson et al*.*^10^. The data are publicly available in the GLODAP repository (https://cchdo.ucsd.edu/, https://www.nodc.noaa.gov/ocads/) and are identified with the expocodes "IcelandSea" and "IrmingerSea". The methodologies have changed slightly over time. A brief summary of these methods is given as follows. From 1983 until the end of 1989, sampling was carried out with water bottles (Nansen-type) equipped with inversion thermometers. Since 1990, a CTD SEA-BIRD (conductivity-temperature-depth) profiler equipped with water bottles in a rosette has been used. The salinity (S) was measured with an Autosal model 8400 salinometer. The oxygen (O_2_) was determined by Winkler titration. Silicate, phosphate and nitrate (nitrate + nitrite) were mainly determined by automatic colorimetric methods and following the QUASIMEME QC programme described in Olafsson et al*.*^10^ with errors less than 1.5% for nitrate, 3.5% for phosphate and 2.5% for silicate. pCO_2_ and DIC were determined following the methods that Dr. Taro Takahashi has been using for more than four decades at LDEO. DIC was determined by coulometry at LDEO until 1991, and until 1993, pCO_2_ was determined by gas chromatography with an overall precision of ± 4 μmol kg^−1^. After these dates, the analyses were carried out at the MRI, where pCO_2_ was determined at a known temperature and pressure using a bubble-type equilibrator system coupled with a gas chromatograph^10^. The evaluation of the CRM analysis at the MRI since 1992 allowed for the correction of biases between 1992–1999 and 2001–2008. The accuracies of the pCO_2_ and DIC measurements were better than ± 2 μatm and ± 2 μmol kg^−1^, respectively^10^. From the DIC and pCO_2_ data, the pH_T_, total alkalinity (TA), and [CO_3_^2−^] were determined using the CO2SYS thermodynamic equations in seawater^56^, the CO_2_ dissociation constants of Lueker et al.^57^. The effect of pressure on the equilibrium constants of CO2SYS is included in the software developed by Lewis & Wallace^58^.

In the IS-TS database, there are 1031 pairs of data with DIC and pCO_2_. In addition, there are 413 unpaired samples with DIC (n = 388) or pCO_2_ (n = 25) with no other carbonate system variable to determine the rest of the variables. Due to the low natural variability of TA, it is the best variable to be computed for these 413 unpaired datasets ^14,27^. We calculated TA using the most recent neural network (NN) technique^59,60^. These NNs compute both TA and DIC from position, T, S, O_2_ and nutrient data. Previously, it was verified that these networks do not produce biases in DIC and TA, comparing the computed values with the measured DIC values or with TA determined from paired DIC and pCO_2_. The differences (calculated–measured) were − 5.6 ± 6.7 and 1.6 ± 5.3 µmol/kg for DIC and TA, respectively, using NN CANYON-B^60^. Finally, a total of 1773 datapoints were calculated using CO2SYS, including the reconstruction of 329 samples where T, S, O_2_ and nutrient measurements are available to use CANYON-B. Ninety-two percent of these samples correspond to non-surface samples taken between 1985 and 1992. For the IRM-TS, the same procedure was applied. From 1689 samples, of which 1628 had T, S, O_2_ and nutrients, 779 pairs with DIC (n = 1246) and pCO_2_ (n = 797) were available. For 485 unpaired samples, TA values were obtained using CANYON-B, completing 1264 data sets with which to compute all the variables of CO2SYS. Then, 364 data points were reconstructed, of which 95% corresponded to non-surface samples obtained from 1983 to 1992 using CANYON-B computing both DIC and TA. This reconstruction was validated by comparing the data produced by CANYON-B against the measured data of DIC (− 2.7 ± 6.7 µmol/kg) and TA (− 0.1 ± 6.4 µmol/kg).

### Variables associated with ocean acidification

The following variables are determined using the database of each station:

1. pH_T_ at in situ conditions is obtained from the pressure, T, S, DIC, TA, silicate and phosphate data using the CO2SYS equations.
2. [H^+^]_T_ in nanomoles per kg of seawater was determined to be 10^(9−pHT)^. Note that changes in pH_T_ represent a relative change in [H^+^]_T_ rather than an absolute change^7,8^.
3. Excess [CO_3_^2−^] (ex[CO_3_^2−^]) is given by [CO_3_^2−^] − K_sp_(Ar)/[Ca^+^] in µmol/kg^3^. This last term is the [CO_3_^2−^] saturation; therefore, positive (negative) values of ex[CO_3_^2−^] indicate oversaturation (undersaturation). This magnitude quantifies the excess or deficit of carbonates over or under saturation, where K_sp_(Ar) is the solubility product of aragonite, which is a non-linear function of temperature, salinity and pressure^61^.

Finally, anthropogenic carbon (C_anth_) was estimated with the biogeochemical back-calculation ϕC_T_° method^62^, which has an overall uncertainty of ± 5.2 μmol kg^−1^. Alternatively, there are two other ways to determine this uncertainty for each layer: (i) based on the time adjustments of C_anth_ (Eq. 2, below) and (ii) by comparison with the determination of C_anth_ using chlorofluorocarbons^63^ (see Supplementary Table S3). Overall, both alternatives estimate uncertainties between 3.1 and 6.1 μmol kg^−1^. The natural fraction in the total DIC (DIC_nat_) is the difference between DIC and C_anth_.

### Layer description and separation

Following the methodology applied at the IS-TS by Jeansson et al*.*^39^, profiles were interpolated for the chosen depths: (i) every 10 m in the upper 300 m, (ii) every 50 m between 300 and 500 m, and (iii) every 100 m from 500 to the bottom. For determining the mixed layer depth (MLD), Jeansson et al*.*^39^ selected the density difference criterion of 0.05 kg/m^3^ after testing several criteria based on differences in temperature and density. This criterion is consistent with early studies^31,64^. Here, this criterion is adopted for both the IS-TS and IRM-TS to define the surface layer. The gradients of T, S and nutrients show a clear attenuation at 300 metres^39^, which allows us to define the subsurface layer from the MLD to a depth of 300 m. Between 300 and 600 m, there is a transition zone with a small vertical gradient^31^, which is considered here as an intermediate layer above the very homogeneous deep layer (600–1900 m). To make a comparative study, the same criteria have been maintained for the IRM-TS, although its oceanographic conditions are different. In Supplementary Fig. S1, the thermohaline characterization of each layer is given by a potential temperature-salinity diagram.

### Detrended time-series

For each layer and mean variable (temperature, S, nutrients, DIC, TA, pH_T_, [H^+^]_T_, ex[CO_3_^2−^] and C_anth_), a multilinear fit is made^65^, including a linear term to determine the long-term trends and two harmonic functions of annual and semi-annual periods to adjust the annual cycle:

1 $$ Y = Y_{{2000}}  + a~\left( {t - 2000} \right) + b_{1} \sin \left( {\frac{{2\Pi \left( {tJ - b_{o} } \right)}}{{365.25}}} \right) + c_{1} \sin \left( {\frac{{4\Pi \left( {tj - c_{o} } \right)}}{{365.25}}} \right) $$

where “*t*” is the time in years and “*tj*” is the Julian day. The two harmonic functions are evaluated by the amplitudes b_1_ and c_1_ with phase and b_o_ and c_o_ on Julian days. After adjustment, the seasonal components are subtracted from the original averaged series of each layer.

2 $$ \Delta Y = Y - Y_{{2000}}  + b_{1} \sin \left( {\frac{{2\Pi \left( {tj - b_{o} } \right)}}{{365.25}}} \right) + c_{1} \sin \left( {\frac{{4\Pi \left( {tj - c_{o} } \right)}}{{365.25}}} \right) $$

By applying this methodology, it is possible to use the complete data series, which is four times more data than using only the winter data, in a homogeneous way in the whole water column. To homogenize the deseasonalized series (ΔY), it is interpolated for Julian days 45, 136, 217 and 316 of each year from 1983 to 2013. These days correspond to the mean Julian days (14-Feb, 15-May, 15-Aug, and 14-Nov) of both series (IS-TS and IRM-TS). Finally, the linear adjustment of ΔY versus '*t*' gives the annual trends (*an_trend*) that are not significantly different from those obtained with Eq. 1 but with better statistical values (r^2^, p-level, and error) due to the reduction of the variability of ΔY in relation to the original series (Y).

### Decomposition of ocean acidification trends

OA trends are induced by changes in T, S, DIC and TA. The impacts of each of these drivers on OA can be identified by breaking down pH_T_, [H^+^]_T_ and ex[CO_3_^2−^] trends^14,27,30,66^ using Taylor decomposition.

3 $$ \frac{{dY}}{{dt}} = \frac{{\partial Y}}{{\partial T}}\frac{{dT}}{{dt}} + \frac{{\partial Y}}{{\partial S}}\frac{{dS}}{{dt}} + \frac{{\partial Y}}{{\partial DIC}}\frac{{dDIC}}{{dt}} + \frac{{\partial Y}}{{\partial TA}}\frac{{dTA}}{{dt}} $$

where the partial derivatives of Y versus T, S, DIC and TA are determined for each layer according to the mean properties for the three Y variables (pH_T_, [H^+^]_T_ and ex[CO_3_^2−^]) using the CO2SYS equations. As changes in DIC and TA and hence changes in pH_T_, [H^+^]_T_ and ex[CO_3_^2−^] can be driven by variations in freshwater fluxes^67^, as well as by variations in ocean internal biogeochemical processes and transport/mixing, it is useful to separate the two mechanisms. We achieve this by expanding the total derivatives and introducing salinity-normalized DIC (nDIC = DIC/S*35) and salinity-normalized Alk (nAlk = Alk/S*35)^66,67^.

4 $$ \frac{{dY}}{{dt}} = \frac{{\partial Y}}{{\partial T}}\frac{{dT}}{{dt}} + \left( {\frac{{\partial Y}}{{\partial S}} + \frac{{\overline{{nDIC}} }}{{S_{o} }}\frac{{\partial Y}}{{\partial DIC}} + \frac{{\overline{{nTA}} }}{{S_{o} }}\frac{{\partial Y}}{{\partial TA}}} \right) \cdot \frac{{dS}}{{dt}} + \frac{{\bar{S}}}{{S_{o} }}\frac{{\partial Y}}{{\partial DIC}}\frac{{dnDIC}}{{dt}} + \frac{{\bar{S}}}{{S_{o} }}\frac{{\partial Y}}{{\partial TA}}\frac{{dnTA}}{{dt}} $$

Now, the new factors of the temporal derivatives of T, S, nDIC and nTA constitute a measure of each of these drivers, which among them do not maintain a direct relation. While the variability of T and S is mainly related to physical processes (warming, freshwater balance and ocean circulation), the variability of nDIC and nTA is linked to biogeochemistry. The processes of synthesis/mineralization of organic matter strongly affect nDIC and weakly affect nTA, while the formation and dissolution of CaCO_3_ affects nTA twice as much as nDIC. Finally, the uncertainty in the calculation of pH as a function of pCO_2_ and DIC (observed variables) is particularly insensitive to the uncertainties of DIC^68^. In contrast, the uncertainty of ex[CO_3_^2−^] is sensitive to both variables^68^, so the uncertainties in relative terms of the ex[CO_3_^2−^] trends are somewhat larger than the pH_T_ (or [H^+^]_T_)_._

## Supplementary Information

Supplementary Information.

## Data Availability

Data were collected and made publicly available by GLODAPv.2.
