# Author Correction: Contrasting drivers and trends of ocean acidification in the subarctic Atlantic

**DOI:** 10.1038/s41598-022-11922-1

**Published:** 2022-05-10

**Authors:** Fiz F. Pérez, Jon Olafsson, Solveig R. Ólafsdóttir, Marcos Fontela, Taro Takahashi

**Affiliations:** 1 Instituto Investigaciones Marinas (IIM, CSIC), Eduardo Cabello, 6, 36208 Vigo, Spain; 2 Institute of Earth Sciences, University of Iceland, Reykjavik, Iceland; 3 Marine and Freshwater Research Institute, Hafnarfjordur, Iceland; 4 Centre of Marine Sciences (CCMAR), Universidade Do Algarve, 8005‑139 Faro, Portugal; 5 Lamont-Doherty Geological Observatory of Columbia University Palisades, Palisades, NY 10964 USA

Correction to: *Scientific Reports* https://doi.org/10.1038/s41598-021-93324-3, published online 07 July 2021

The original version of this Article contained errors.

In Table 2 legend, the symbol of “picomol” was incorrectly given as “nanomol”.

“Average trends obtained with the seasonally detrended data the in situ temperature (T in °C yr^−1^), salinity (S in yr^−1^), Total Alkalinity (TA in µmol kg^−1^ yr^−1^), salinity-normalized alkalinity (nTA in µmol kg^−1^ yr^−1^), total dissolved inorganic carbon (DIC in µmol kg^−1^ yr^−1^), salinity-normalized dissolved inorganic carbon (nDIC in µmol kg^−1^ yr^−1^), in situ pH in total scale (pHT yr^−1^), total hydrogen ion concentrations ([H+]T in nanomol kg^−1^ yr^−1^), ion carbonate concentration excess over aragonite saturation (exCO_3_ = in µmol kg^−1^ yr^−1^), and anthropogenic CO_2_.”

now reads:

“Average trends obtained with the seasonally detrended data the in situ temperature (T in °C yr^−1^), salinity (S in yr^−1^), Total Alkalinity (TA in µmol kg^−1^ yr^−1^), salinity-normalized alkalinity (nTA in µmol kg^−1^ yr^−1^), total dissolved inorganic carbon (DIC in µmol kg^−1^ yr^−1^), salinity-normalized dissolved inorganic carbon (nDIC in µmol kg^−1^ yr^−1^), in situ pH in total scale (pHT yr^−1^), total hydrogen ion concentrations ([H+]T in picomol kg^−1^ yr^−1^), ion carbonate concentration excess over aragonite saturation (exCO_3_ = in µmol kg^−1^ yr^−1^), and anthropogenic CO_2_.”

Additionally, the article contains a repeated error where the symbol for “pmol” was incorrectly given as “nmol” in the Results section, under the subheading ‘Acidifcation drivers’, in Figure 6 legend, and in the Conclusions.

Furthermore, in Figure 6A and Supplementary Figure S5A “pmol” was incorrectly given as “nmol” in the y-axis. The original Figure 6 and accompanying legend, and Supplementary Information file appear below.

**Figure 6:**
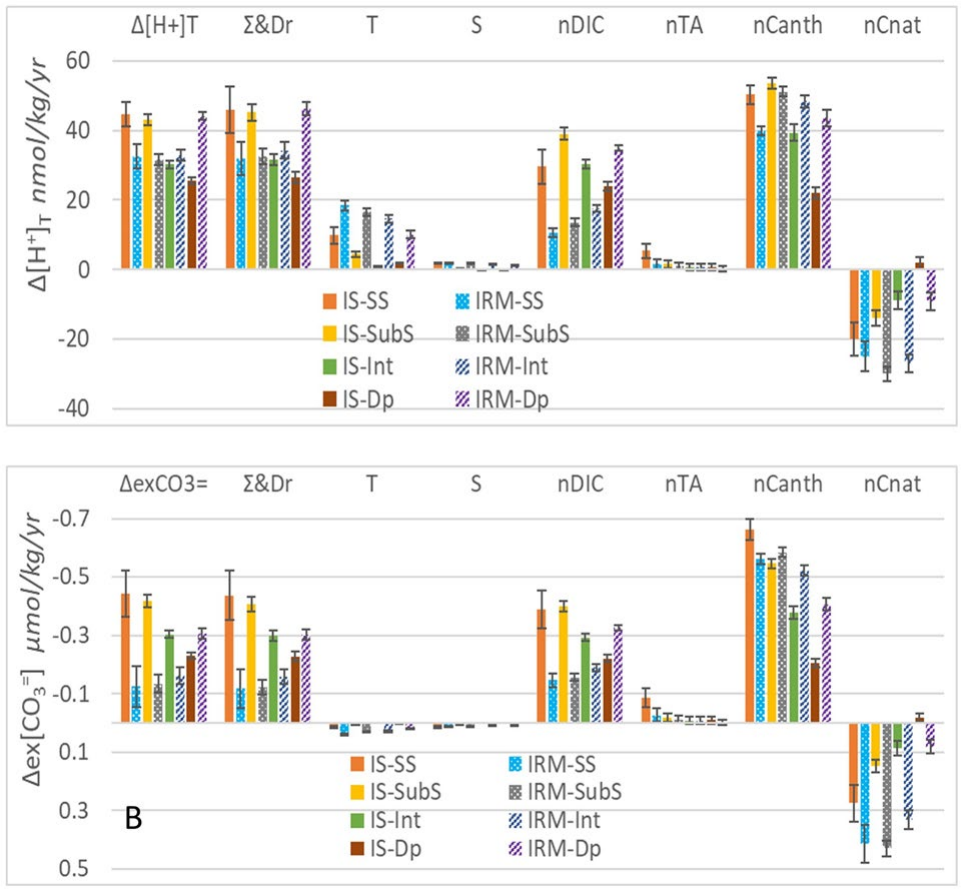
Acidification trends and drivers decomposition (T,S, nDIC and nTA) for the seasonally detrended average time series of total hydrogen ions concentration in pmol/kg/yr (Δ**[H**^**+**^**]**_**T**_, **A**) and for excess of [CO_3_^=^ ] over the [CO_3_^=^ ] at aragonite saturation in µmol/kg/yr (Δ**ex[CO**_**3**_^**=**^**]**, **B**). The nDIC driver trends is split in natural (nCnat) and anthropogenic components (nCanth). The colour code is shown on both panels.

The original Article and accompanying Supplementary Information file have been corrected.

## Supplementary Information

Supplementary Information.

