# Supplementary material for: Contrasting drivers and trends of ocean acidification in the subarctic Atlantic
Source: Sci Rep. 2021 Jul 7;11:13991. doi: 10.1038/s41598-021-93324-3 (PMC8263757; doi:10.1038/s41598-021-93324-3)
Supplement: Supplementary file 1 — Supplementary Information. [file 41598_2021_93324_MOESM1_ESM.docx]

Supplementary Information of

**Contrasting drivers and trends of ocean acidification in the subarctic Atlantic**

Fiz F. Pérez^1*^, Jon Olafsson^2^, Solveig R. Ólafsdóttir^3^, Marcos Fontela^4^, Taro Takahashi^5^

^1^Instituto Investigaciones Marinas (IIM, CSIC), Eduardo Cabello, 6, 36208, Vigo, Spain

^2^Institute of Earth Sciences, University of Iceland, Reykjavik, Iceland

^3^Marine and Freshwater Research Institute, Hafnarfjordur, Iceland

^4^Centre of Marine Sciences (CCMAR), Universidade do Algarve, 8005-139 Faro, Portugal.

^5^Lamont-Doherty Geological Observatory of Columbia University Palisades, NY 10964, USA

^5^ Deceased

^*^Corresponding author: Fiz F. Pérez ([fiz.perez@iim.csic.es](mailto:fiz.perez@iim.csic.es))

| 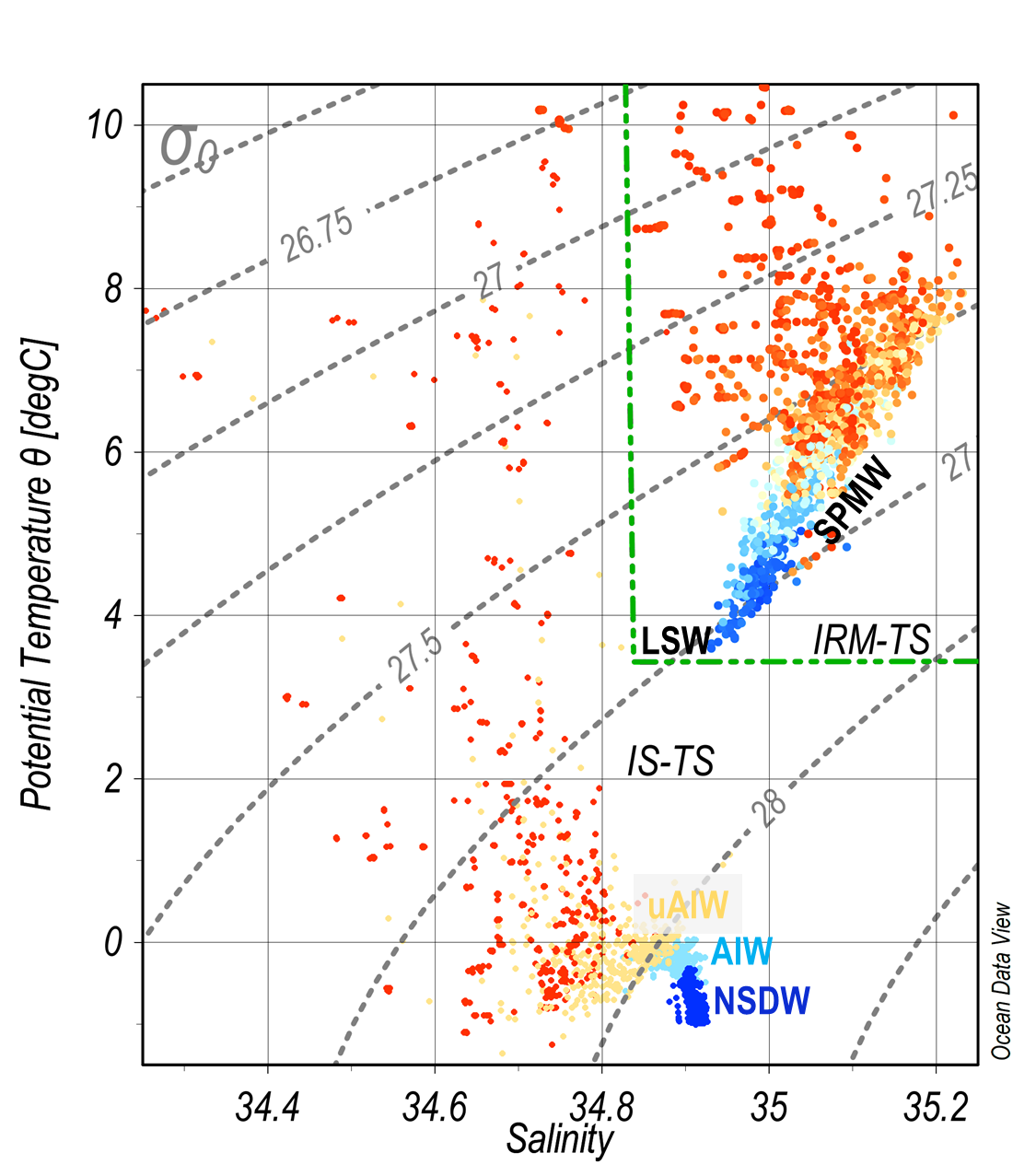 |
| --- |
| *Figure S1.-* Theta-S diagram showing the main water masses involved in IS-TS and IRM-TS (inlet). Color codes show surface (red; surface to MLD), subsurface (yellow; MLD-200 meters), intermediate (cyan; 200-600 meters) and Deep (dark blue; 600 meters to bottom) layer. For IS-TS, below surface layer, the main water masses are upper Arctic Intermediate Water (AIW), AIW and Norwegian Sea Deep Water (NSDW) following Swift and Aagaard (1981). For IRM-TS, below surface layer, the main water masses different varieties of SubPolar Mode Water mixing with LSW (Labrador Sea Water) following (Garcia-Ibañez et al. 2015). |

| 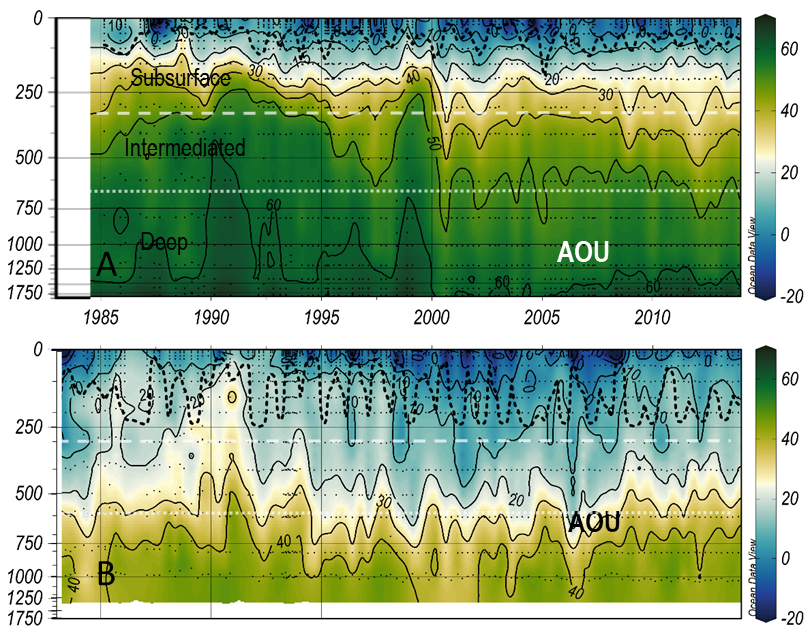 |
| --- |
| *Figure S2.- Vertical and time variation from 1983 (1985 for IS-TS) to 2013 of Apparent Oxygen Utilization (AOU) in µmol/kg at IS-TS (A) and at IRM-TS (B) in color scales and black isolines. Dash black line is showing the mixed layer depth that delimits the surface from the subsurface layers.* *The dashed black line shows the mixed layer depth that delimits the surface from the subsurface layers. The white dashed line separates the subsurface and intermediate layers, and the white dotted lines separate the intermediate and deep layers (labelled in A).**AOU is the oxygen saturation, calculated using potential temperature and salinity, less the measured oxygen. Figure prepared using Ocean Data View/DIVA* *Schlitzer, R., 2020. Ocean Data View. http://odv.awi.de* |

| 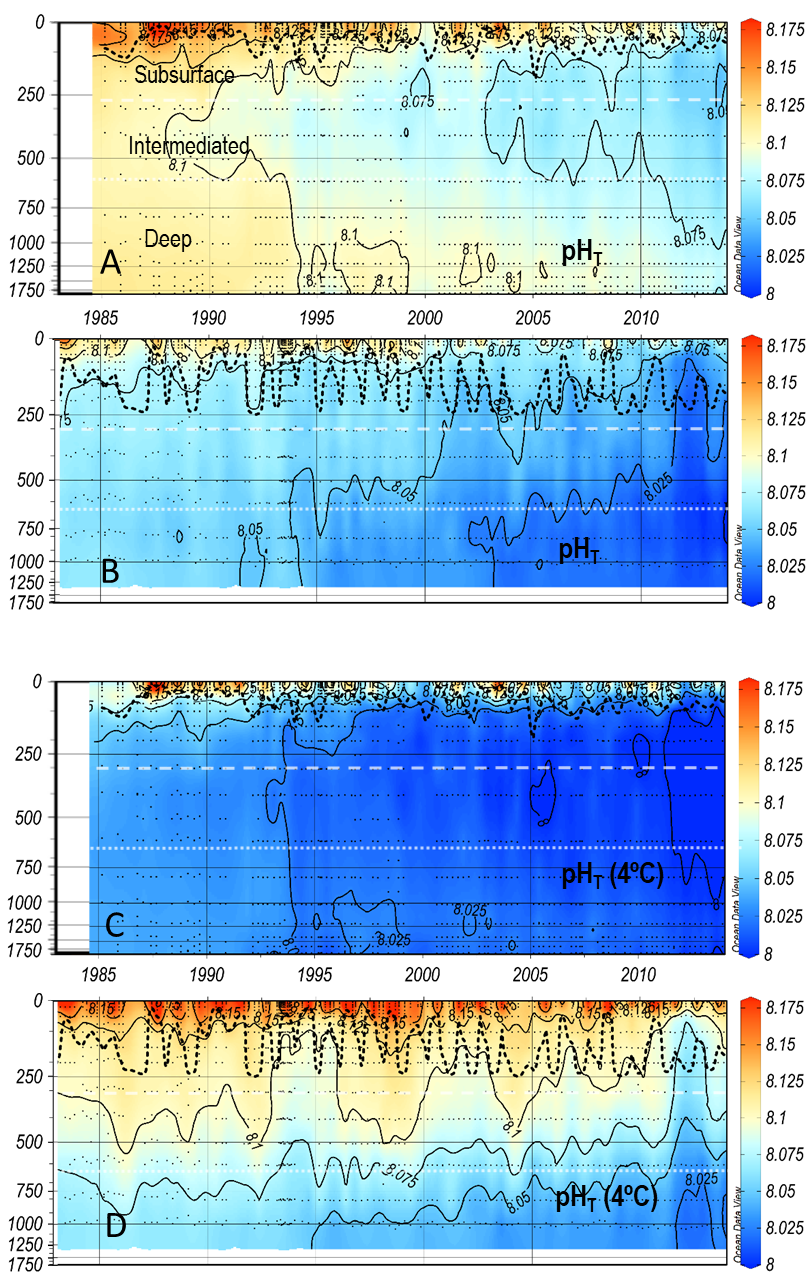 |
| --- |
| *Figure S3.- As figure S2 for pH_T_ and pH_T_  referred to 4ºC at IS-TS (A and C) and at IRM-TS (B and D) in black isolines and colour. Figure prepared using Ocean Data View/DIVA* *Schlitzer, R., 2020. Ocean Data View. http://odv.awi.de* |

| 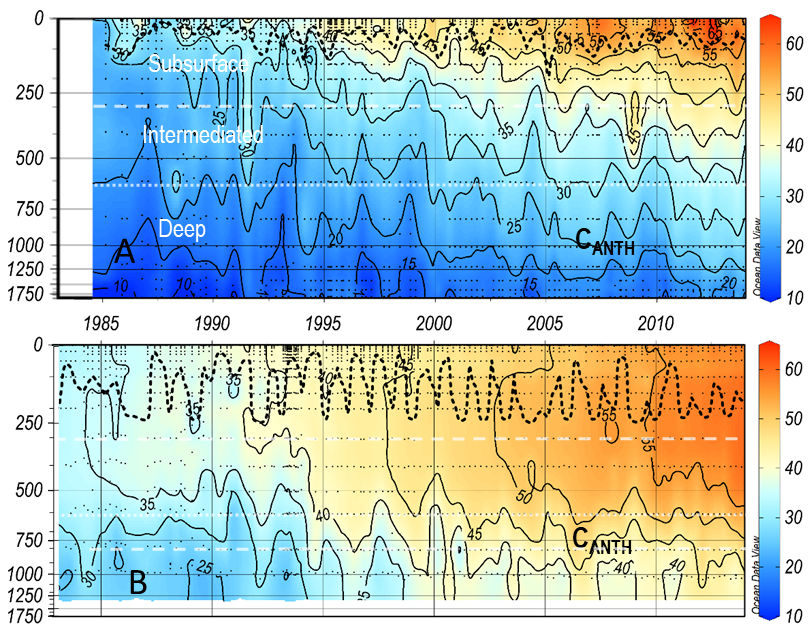 |
| --- |
| *Figure S4.- As figure S2 for C_ANTH_ at IS-TS (A) and at IRM-TS (B). Figure prepared using Ocean Data View/DIVA* *Schlitzer, R., 2020. Ocean Data View. http://odv.awi.de* |

| 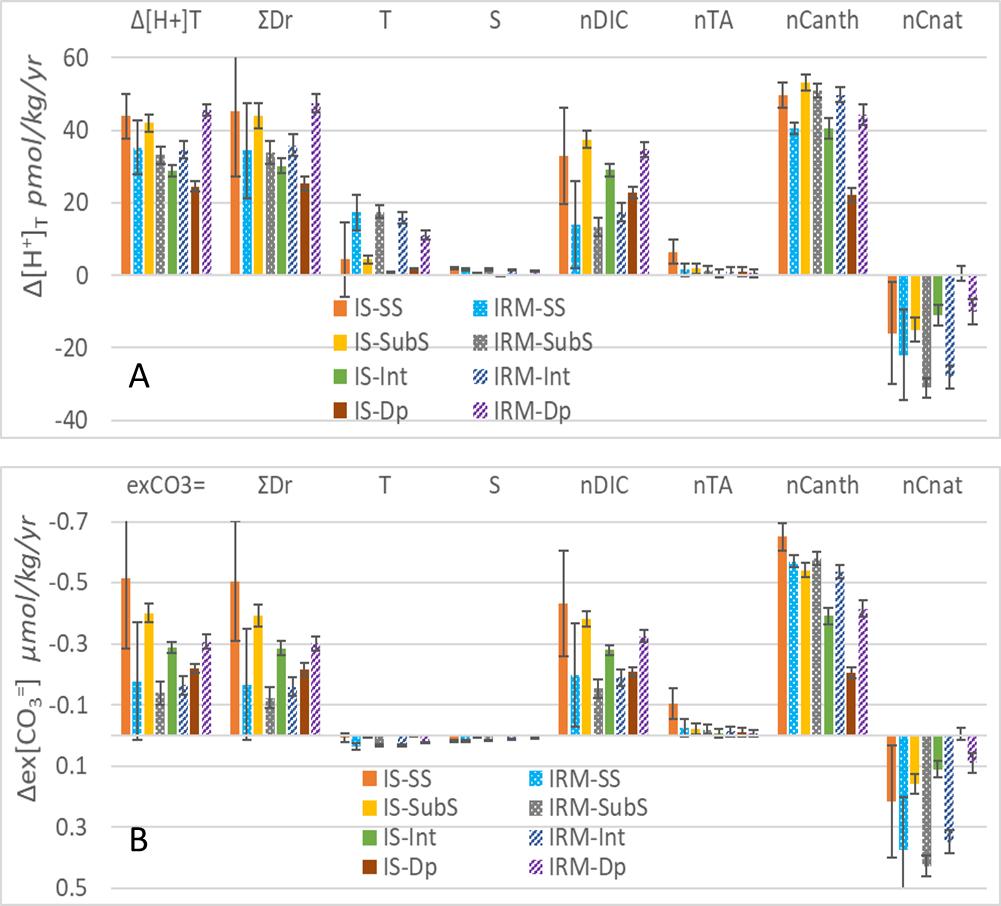 |
| --- |
| *Figure S5.-* Acidification trends and drivers decomposition (T,S, nDIC and nTA) for total hydrogen ions concentration in pmol/kg/yr (Δ**[H^+^]_T_** , A) and for excess of [CO_3_^=^ ] over the [CO_3_^=^ ] at aragonite saturation in µmol/kg/yr (Δ**ex[CO_3_^=^]**, B). The nDIC driver trends is split in natural (nCnat) and anthropogenic components (nCanth). The colour code is shown on both panels. The error bar is including. All trends are computed using the original data. In figure 6 the trends are computed from the detrended time-series. |

Table S1. Average trends obtained for pH (10^-3^ yr^-1^), aragonite saturation (Ωar, 10^-3^ yr^-1^), temperature (T in ºC), salinity (S), dissolved inorganic carbon (DIC in µmol·kg^-1^·yr^-1^), Total Alkalinity (TA in µmol·kg^-1^·yr^-1^), anthropogenic CO_2_, (C_anth_ in µmol·kg^-1^·yr^-1^) using only winter data, all data, and the seasonally detrended data at the Iceland Sea station (IS-TS). Included for the four layers defined in Methods are slopes and their error, as well as p-level and R^2^ (in parenthesis) in intercalated rows.

| Layer | Method | pH*10^3^ | Ωar*10^3^ | T | S | TA | DIC | Cant |
| --- | --- | --- | --- | --- | --- | --- | --- | --- |
| **Surface** |  |  |  |  |  |  |  |  |
| trend | **Winter** | -2.84±0.20 | -6.72±1.07 | 0.036±0.010 | 0.0039±0.0018 | 0.17±0.10 | 1.00±0.13 | 1.19±0.08 |
| p-level (R^2^) | n=27 | 0.0050 (0.89) | 0.02 (0.61) | 0.08 (0.33) | 0.18 (0.16) | 0.30 (0.10) | 0.018 (0.69) | 0.0047 (0.90) |
| Trend | **All data** | *-2.54±0.37* | *-7.70±3.51* | *0.016±0.036* | *0.0050±0.0011* | *0.16±0.08* | *1.05±0.33* | *1.13±0.08* |
| p-level (R^2^) | n=109 | *0.02 (0.30)* | *0.19 (0.04)* | *0.99 (0.00)* | *0.05 (0.16)* | *0.25 (0.03)* | *0.09 (0.09)* | *0.0050 (0.65)* |
| Trend | **Deseasonalized** | **-2.58±0.22** | **-6.71±1.17** | **0.034±0.009** | **0.0044±0.0008** | **0.156±0.076** | **0.94±0.13** | **1.15±0.06** |
| p-level (R^2^) | n=109 | **0.0070 (0.56)** | **0.03 (0.23)** | **0.06 (0.12)** | **0.03 (0.21)** | **0.17 (0.046)** | **0.02 (0.31)** | **0.0029 (0.75)** |
| **Subsurface** |  |  |  |  |  |  |  |  |
| Trend | **Winter** | -2.25±0.15 | -6.10±0.59 | 0.010±0.004 | 0.0007±0.0006 | 0.01±0.06 | 0.78±0.06 | 0.96±0.07 |
| p-level (R^2^) | n=27 | 0.0044 (0.90) | 0.0093 (0.81) | 0.12 (0.24) | 0.53 (0.05) | 1.00 (0.00) | 0.005 (0.89) | 0.0049 (0.89) |
| Trend | **All data** | *-2.20±0.12* | *-5.84±0.49* | *0.014±0.004* | *0.0012±0.0004* | *0.04±0.04* | *0.77±0.05* | *0.98±0.04* |
| p-level (R^2^) | n=109 | *0.0032 (0.74)* | *0.0069 (0.57)* | *0.06 (0.13)* | *0.09 (0.10)* | *0.47 (0.01)* | *0.0046 (0.67)* | *0.0018 (0.84)* |
| Trend | **Deseasonalized** | **-2.26±0.08** | **-6.02±0.29** | **0.014±0.002** | **0.0010±0.0003** | **0.034±0.03** | **0.78±0.03** | **1.00±0.03** |
| p-level (R^2^) | n=109 | **0.0013 (0.87)** | **0.0023 (0.79)** | **0.03 (0.22)** | **0.09 (0.09)** | **0.67 (0.01)** | **0.0019 (0.82)** | **0.0010 (0.90)** |
| **Intermediate** |  |  |  |  |  |  |  |  |
| Trend | **Winter** | -1.65±0.12 | -4.53±0.38 | 0.003±0.002 | -0.0003±0.0002 | -0.03±0.04 | 0.56±0.05 | 0.77±0.09 |
| p-level (R^2^) | n=27 | 0.0054 (0.88) | 0.0068 (0.85) | 0.33 (0.09) | 0.31 (0.10) | 0.79 (0.02) | 0.007 (0.85) | 0.0130 (0.75) |
| Trend | **All data** | *-1.46±0.08* | *-4.00±0.23* | *0.002±0.001* | *-0.0002±0.0001* | *-0.03±0.03* | *0.50±0.03* | *0.72±0.05* |
| p-level (R^2^) | n=109 | *0.0029 (0.76)* | *0.0033 (0.74)* | *0.14 (0.06)* | *0.24 (0.03)* | *0.63 (0.01)* | *0.0034 (0.73)* | *0.0050 (0.65)* |
| Trend | **Deseasonalized** | **-1.52±0.06** | **-4.12±0.17** | **0.002±0.001** | **-0.0003±0.0001** | **-0.043±0.02** | **0.52±0.02** | **0.70±0.04** |
| p-level (R^2^) | n=109 | **0.0016 (0.84)** | **0.0016 (0.85)** | **0.12 (0.06)** | **0.08 (0.10)** | **0.30 (0.02)** | **0.0019 (0.82)** | **0.0033 (0.73)** |
| **Deep** |  |  |  |  |  |  |  |  |
| Trend | **Winter** | -1.25±0.10 | -2.75±0.26 | 0.005±0.001 | -0.00038±0.00008 | -0.03±0.05 | 0.41±0.03 | 0.41±0.07 |
| p-level (R^2^) | n=27 | 0.0070 (0.85) | 0.0089 (0.82) | 0.0105 (0.79) | 0.04 (0.47) | 0.94 (0.01) | 0.007 (0.85) | 0.02 (0.62) |
| Trend | **All data** | *-1.18±0.07* | *-2.56±0.16* | *0.005±0.000* | *-0.00025±0.00005* | *-0.03±0.02* | *0.37±0.03* | *0.38±0.03* |
| p-level (R^2^) | n=109 | *0.0031 (0.75)* | *0.0038 (0.71)* | *0.0015 (0.86)* | *0.04 (0.19)* | *0.35 (0.02)* | *0.005 (0.63)* | *0.0079 (0.54)* |
| Trend | **Deseasonalized** | **-1.23±0.05** | **-2.66±0.10** | **0.005±0.000** | **-0.0003±0.0000** | **-0.04±0.02** | **0.39±0.02** | **0.38±0.03** |
| p-level (R^2^) | n=109 | **0.0016 (0.85)** | **0.0015 (0.85)** | **0.0015 (0.86)** | **0.01 (0.41)** | **0.23 (0.03)** | **0.0029 (0.82)** | **0.0050 (0.64)** |

Table S2. The same as Table S1 for Irminger Station (IRM-TS)

| Layer | Method | pH*10^3^ | Ωar*10^3^ | T | S | TA | DIC | Cant |
| --- | --- | --- | --- | --- | --- | --- | --- | --- |
| **Surface** |  |  |  |  |  |  |  |  |
| trend | Winter | -1.48±0.18 | -1.22±1.17 | 0.058±0.009 | 0.004±0.001 | 0.24±0.10 | 0.44±0.06 | 0.84±0.07 |
| p-level (R^2^) | n=30 | 0.0140 (0.72) | 0.60 (0.03) | 0.03 (0.58) | 0.04 (0.45) | 0.17 (0.16) | 0.02 (0.63) | 0.0071 (0.83) |
| Trend | All data | *-1.87±0.42* | *-2.67±2.99* | *0.059±0.017* | *0.004±0.001* | *0.25±0.06* | *0.59±0.29* | *0.96±0.04* |
| p-level (R^2^) | n=124 | *0.05 (0.14)* | *0.71 (0.01)* | *0.08 (0.09)* | *0.03 (0.21)* | *0.05 (0.13)* | *0.22 (0.03)* | *0.0014 (0.86)* |
| Trend | Deseasonalized | **-1.73±0.20** | **-1.81±1.08** | **0.063±0.005** | **0.004±0.001** | **0.22±0.04** | **0.49±0.10** | **0.93±0.03** |
| p-level (R^2^) | n=124 | **0.0127 (0.39)** | **0.30 (0.02)** | **0.0060 (0.58)** | **0.02 (0.30)** | **0.03 (0.19)** | **0.04 (0.16)** | **0.0009 (0.90)** |
| **Subsurface** |  |  |  |  |  |  |  |  |
| Trend | Winter | -1.29±0.20 | -0.58±1.04 | 0.057±0.009 | 0.004±0.001 | 0.18±0.09 | 0.32±0.07 | 0.89±0.09 |
| p-level (R^2^) | n=30 | 0.02 (0.59) | 0.96 (0.01) | 0.02 (0.61) | 0.04 (0.47) | 0.20 (0.14) | 0.05 (0.41) | 0.0100 (0.78) |
| Trend | All data | *-1.59±0.11* | *-2.08±0.61* | *0.053±0.005* | *0.004±0.001* | *0.22±0.04* | *0.49±0.04* | *1.00±0.04* |
| p-level (R^2^) | n=124 | *0.0050 (0.62)* | *0.08 (0.09)* | *0.0093 (0.47)* | *0.0151 (0.35)* | *0.03 (0.22)* | *0.007 (0.54)* | *0.0014 (0.86)* |
| Trend | Deseasonalized | **-1.53±0.08** | **-2.01±0.45** | **0.050±0.003** | **0.004±0.000** | **0.22±0.03** | **0.49±0.03** | **1.00±0.03** |
| p-level (R^2^) | n=124 | **0.0026 (0.76)** | **0.05 (0.14)** | **0.0044 (0.65)** | **0.012 (0.40)** | **0.021(0.28)** | **0.0037 (0.69)** | **0.0008 (0.91)** |
| **Intermediate** |  |  |  |  |  |  |  |  |
| Trend | Winter | -1.39±0.20 | -1.57±0.98 | 0.047±0.009 | 0.003±0.001 | 0.11±0.09 | 0.36±0.07 | 0.77±0.08 |
| p-level (R^2^) | n=30 | 0.0199 (0.64) | 0.32 (0.08) | 0.03 (0.50) | 0.05 (0.39) | 0.53 (0.04) | 0.03 (0.52) | 0.0104 (0.77) |
| Trend | All data | *-1.61±0.10* | *-2.24±0.46* | *0.047±0.004* | *0.003±0.000* | *0.18±0.03* | *0.52±0.04* | *0.93±0.04* |
| p-level (R^2^) | n=124 | *0.0041 (0.67)* | *0.04 (0.16)* | *0.0085 (0.49)* | *0.0154 (0.35)* | *0.04 (0.18)* | *0.0058 (0.59)* | *0.0018 (0.82)* |
| Trend | Deseasonalized | **-1.54±0.07** | **-2.26±0.40** | **0.043±0.003** | **0.003±0.000** | **0.18±0.03** | **0.51±0.03** | **0.90±0.03** |
| p-level (R^2^) | n=124 | **0.0023 (0.79)** | **0.03 (0.21)** | **0.0058 (0.59)** | **0.011 (0.42)** | **0.03 (0.24)** | **0.0034 (0.71)** | **0.0012 (0.88)** |
| **Deep** |  |  |  |  |  |  |  |  |
| Trend | Winter | -1.96±0.13 | -3.97±0.67 | 0.028±0.009 | 0.002±0.001 | 0.12±0.07 | 0.70±0.06 | 0.68±0.11 |
| p-level (R^2^) | n=30 | 0.0044 (0.89) | 0.03 (0.54) | 0.10 (0.25) | 0.07 (0.32) | 0.30 (0.09) | 0.0065 (0.85) | 0.02 (0.59) |
| Trend | All data | *-2.01±0.07* | *-3.95±0.30* | *0.031±0.004* | *0.002±0.000* | *0.13±0.03* | *0.71±0.03* | *0.74±0.05* |
| p-level (R^2^) | n=124 | *0.0014 (0.86)* | *0.0058 (0.59)* | *0.0145 (0.36)* | *0.016 (0.34)* | *0.04 (0.16)* | *0.0016 (0.83)* | *0.0044 (0.65)* |
| Trend | Deseasonalized | **-1.95±0.04** | **-3.98±0.23** | **0.028±0.003** | **0.002±0.000** | **0.13±0.02** | **0.71±0.02** | **0.73±0.04** |
| p-level (R^2^) | n=124 | **0.0005 (0.94)** | **0.0035 (0.70)** | **0.012 (0.42)** | **0.012 (0.41)** | **0.03 (0.23)** | **0.0009 (0.90)** | **0.0029 (0.74)** |

Table S3. Average and standard deviations of the differences between the anthropogenic CO_2_ (ΔC_anth_ µmol·kg^-1^) measured and those fitted by the equation 2 in Methods. These standard deviations could consider an upward measure of the uncertainty of the C_anth_ estimation because the equation 2 fit for almost the temporal variability of the layer averaged C_anth_ although not all the times scales are including such as interannual or decadal variability. The column “C_anth_ −TTD-C_anth_” shows the differences between the C_anth_ computed here and the one determined with the TTD method based on CFC-12 measurements by Lauset et al. 2016. Both data sets show a high correlation (r2=0.89 n=1325). The differences show no temporal correlation (r^2^=0.001, plevel=0.48), but a slight correlation with depth (r2=0.036, plevel=0.02) of 0.0015 µmol·kg^-1^ m^-1^. Finally, the two columns on the right show the trends calculated using the TTD-C_anth_ estimates and those obtained here by the back-calcution method.

| **Layer** | St. | ΔC_anth_ | C_anth_ −TTD-C_anth_ | TTD-C_anth_ Trends | C_anth_ Trends |
| --- | --- | --- | --- | --- | --- |
| **Surface** | **IS** | **6.1** | **3±5.3** (528) | **0.99±0.02** | **1.15 ±0.06** |
| 0 to MLD | IRM | 3.3 | − | − | − |
| **Subsurface** | **IS** | **3.1** | **0.3±4.3** (148) | **0.88±0.02** | **1.00±0.03** |
| MLD to 300 m | IRM | 3.5 | − | − | − |
| **Intermediate** | **IS** | **4.0** | −**0.4±3.8** (131) | **0.73±0.01** | **0.70±0.04** |
| 300- 600 m | IRM | 3.7 | − | − | − |
| **Deep** | **IS** | **4.0** | −**0.2±3.3** (505) | **0.44±0.01** | **0.38±0.01** |
| 600- bottom | IRM | 4.7 | − | − | − |
| Mean | **IS** | **4.0** | **1.1±4.6** (1324) |  |  |
| Mean | IRM | 3.8 | − |  |  |
